# A Phase 1, Placebo-controlled, Randomized, Single Ascending Dose Study and a Volunteer Infection Study to Characterize the Safety, Pharmacokinetics, and Antimalarial Activity of the *Plasmodium* Phosphatidylinositol 4-Kinase Inhibitor MMV390048

**DOI:** 10.1093/cid/ciaa368

**Published:** 2020-04-02

**Authors:** James S McCarthy, Cristina Donini, Stephan Chalon, John Woodford, Louise Marquart, Katharine A Collins, Felix D Rozenberg, David A Fidock, Mohammed H Cherkaoui-Rbati, Nathalie Gobeau, Jörg J Möhrle

**Affiliations:** 1 QIMR Berghofer Medical Research Institute, Brisbane, Queensland, Australia; 2 Medicines for Malaria Venture, Geneva, Switzerland; 3 Department of Microbiology and Immunology, Columbia University Irving Medical Center, New York, New York, USA; 4 Division of Infectious Diseases, Department of Medicine, Columbia University Irving Medical Center, New York, New York, USA

**Keywords:** MMV390048, pharmacokinetics, safety, antimalarial, *Plasmodium* phosphatidylinositol 4-kinase

## Abstract

**Background:**

MMV390048 is the first *Plasmodium* phosphatidylinositol 4-kinase inhibitor to reach clinical development as a new antimalarial. We aimed to characterize the safety, pharmacokinetics, and antimalarial activity of a tablet formulation of MMV390048.

**Methods:**

A 2-part, phase 1 trial was conducted in healthy adults. Part 1 was a double-blind, randomized, placebo-controlled, single ascending dose study consisting of 3 cohorts (40, 80, 120 mg MMV390048). Part 2 was an open-label volunteer infection study using the *Plasmodium falciparum* induced blood-stage malaria model consisting of 2 cohorts (40 mg and 80 mg MMV390048).

**Results:**

Twenty four subjects were enrolled in part 1 (n = 8 per cohort, randomized 3:1 MMV390048:placebo) and 15 subjects were enrolled in part 2 (40 mg [n = 7] and 80 mg [n = 8] cohorts). One subject was withdrawn from part 2 (80 mg cohort) before dosing and was not included in analyses. No serious or severe adverse events were attributed to MMV390048. The rate of parasite clearance was greater in subjects administered 80 mg compared to those administered 40 mg (clearance half-life 5.5 hours [95% confidence interval {CI}, 5.2–6.0 hours] vs 6.4 hours [95% CI, 6.0–6.9 hours]; *P* = .005). Pharmacokinetic/pharmacodynamic modeling estimated a minimum inhibitory concentration of 83 ng/mL and a minimal parasiticidal concentration that would achieve 90% of the maximum effect of 238 ng/mL, and predicted that a single 120-mg dose would achieve an adequate clinical and parasitological response with 92% certainty.

**Conclusions:**

The safety, pharmacokinetics, and pharmacodynamics of MMV390048 support its further development as a partner drug of a single-dose combination therapy for malaria.

**Clinical Trials Registration:**

NCT02783820 (part 1); NCT02783833 (part 2).

## Teaser

MMV390048 is a promising new antimalarial compound in clinical development. We characterized the safety, pharmacokinetics, and antimalarial activity of MMV390048 in healthy adults. The results support the development of MMV390048 as a partner drug of a single-dose combination malaria therapy.

Drug resistance has emerged against all available antimalarial drugs [1], threatening the goal of malaria elimination articulated by the World Health Organization (WHO) [2]. New drugs are needed to combat drug-resistant parasites, reduce malaria morbidity and mortality, and facilitate the ultimate elimination of the disease. New antimalarial therapies will ideally have novel modes of action, and will cure patients after a single administration, due to compliance challenges with multidose treatments.

Several antimalarial candidates are currently in clinical development [3]. One of these is MMV390048, the first *Plasmodium* phosphatidylinositol 4-kinase (PI4K) inhibitor to reach clinical development [4]. PI4K is a particularly attractive target for antimalarial drug development because it is required across all *Plasmodium* lifecycle stages [5]. A powder-in-bottle and 2 tablet formulations (tartaric acid and Syloid) of MMV390048 have been tested in 3 phase 1 studies [6]. MMV390048 was well tolerated in healthy subjects up to a single dose of 120 mg. The tablet formulations had the least intersubject pharmacokinetic (PK) variability, indicating potential for antimalarial prophylaxis or as a single-dose cure.

This communication describes a 2-part phase 1 trial where the safety, tolerability, PK, and antimalarial activity of the tartaric acid tablet formulation of MMV390048 were characterized. The first part was a randomized single ascending dose (SAD) study where a single dose of MMV390048 or placebo was administered to healthy subjects. The second part was a volunteer infection study (VIS) using the induced blood-stage malaria (IBSM) model [7] in which healthy malaria-naive subjects were inoculated with *Plasmodium falciparum* and subsequently treated with a single dose of MMV390048. The antimalarial activity of MMV390048 was assessed, and the pharmacokinetic/pharmacodynamic (PK/PD) relationship was modeled to estimate the curative dose in clinical malaria.

## METHODS

### Study Design and Participants

Part 1 was a double-blind, randomized, placebo-controlled, parallel group, SAD study. Part 2 was an open-label VIS using the *P. falciparum* IBSM model. Healthy males and females of nonchildbearing potential, aged 18–55 years, were eligible for inclusion in both parts; subjects in the VIS were malaria naive (inclusion and exclusion criteria are listed in the Supplementary Materials). The study was conducted at Q-Pharm (Brisbane, Australia) following approval by the QIMR Berghofer Human Research Ethics Committee. All subjects gave written informed consent before enrollment. The study was registered at ClinicalTrials.gov (NCT02783820 [part 1]; NCT02783833 [part 2]).

### Randomization and Blinding

Subjects in the SAD study were randomized within each dose cohort to either MMV390048 or placebo in a 3:1 ratio. Subjects and investigators were blind to the identity of the study intervention from the time of randomization until database lock. The identity of the study intervention was concealed by identical packaging, appearance, odor, and taste. Randomization schedules were generated electronically by independent, unblinded statisticians using the PROC PLAN procedure in SAS software.

### Procedures

The SAD study was conducted in 3 dose cohorts (40, 80, and 120 mg). Subjects received a single dose of MMV390048 or placebo tablets (Corealis Pharma, Canada) after fasting for at least 4 hours; no food was allowed until 4 hours postdosing. Subjects were confined to the clinic for 48 hours and then monitored as outpatients until the end of the study (day 28).

The VIS was conducted in 2 dose cohorts (40 and 80 mg). Subjects were inoculated intravenously on day 0 with *P. falciparum*–infected erythrocytes (~2800 viable parasites) [7] with parasitemia monitored by quantitative polymerase chain reaction (qPCR) targeting the *P. falciparum* 18S ribosomal RNA gene [8]. A single dose of MMV390048 tablets was administered fasted when parasitemia reached approximately 5000 parasites/mL. The doses of MMV390048 selected were predicted to be subtherapeutic based on preclinical modeling [4] and PK data obtained in previous clinical studies [6], and thus chosen to facilitate modeling to estimate the PK/PD relationship. Subjects received a standard course of artemether/lumefantrine 25 days postdosing, or earlier if recrudescence occurred. Gametocytemia was monitored by quantitative reverse-transcription PCR (qRT-PCR) targeting a messenger RNA transcript of mature female gametocytes, *pfs25* [9]. If gametocytes were detected at the time of treatment with artemether/lumefantrine, a single 45-mg dose of primaquine was administered. Subjects were monitored until the end of the study (days 43–62).

### Outcomes

#### Safety

Safety assessments included adverse event (AE) recording, physical examinations, vital signs monitoring, electrocardiography, and clinical laboratory evaluation (hematology, biochemistry, urinalysis). Additionally, inhibin B, follicle-stimulating hormone, luteinizing hormone, and testosterone were measured for endocrine assessment of testicular function (SAD only) since testicular toxicity was observed in 1 of the 2 preclinical toxicology species (rats but not dogs). AE severity was assessed in accordance with the WHO Handbook for Reporting of Results of Cancer Treatment (SAD) or the Common Terminology Criteria for Adverse Events version 4.03 (VIS).

#### Pharmacokinetics

Blood was collected to determine the MMV390048 plasma concentration using liquid chromatography–tandem mass spectrometry [6]. Samples were collected predose and at the following timepoints after dosing: 0.5, 1, 2, 3, 4, 6, 8, 12, 24, 48, 96, and 144 hours (day 6), 216 hours (day 9), 312 hours (day 13), 432 hours (day 18), 600 hours (day 25), and 672 hours (day 28; end of study). Noncompartmental PK analysis was performed using Phoenix WinNonlin Pro version 6.3 (Certara LP, St Louis, Missouri). Pharmacokinetic endpoints were peak plasma concentration (C_max_), timepoint at which C_max_ is reached (T_max_), area under the concentration-time curve from 0 hour to the last measured timepoint (AUC_0-last_), area under the concentration-time curve from 0 hour to infinity (AUC_0-∞_), estimated elimination phase half-life (T_1/2_), apparent clearance (CL/F), and apparent volume of distribution (Vz/F).

#### Pharmacodynamics

Blood was collected for qPCR predose and at the following timepoints after dosing: 2, 4, 8, 12, 16, 20, 24, 30, 36, 48, 60, 72, and 84 hours. The PD variable of interest was the rate of parasite clearance as measured by the parasite reduction ratio (PRR) and parasite clearance half-life, with the former expressed as the log_10_ transformed ratio of the parasite density decrease over a 48-hour time period (log_10_PRR_48_) [10]. Differences in log_10_PRR_48_ between cohorts were determined using an omnibus test [11]. Analysis was performed in R software (version 3.3.0).

#### PK/PD Modeling

To maximize model accuracy, PK/PD modeling included data from 3 previous clinical studies of MMV390048 [6] in addition to data obtained in the current study (Supplementary Table 1). The demographic characteristics of subjects included in the PK/PD datasets are summarized in Supplementary Table 2 and Supplementary Table 3. Modeling was performed in R software (Microsoft Open R, version 3.4.3) combined to the IQR Tools package from IntiQuan, Basel, Switzerland (version 0.7.2). Parameters were estimated by nonlinear mixed-effect models in Monolix (version 2016R1). Endpoints were the minimum inhibitory concentration (MIC) and the minimum parasiticidal concentration that achieves 90% of the maximum effect (MPC_90_). The efficacious single dose of MMV390048 (the dose that would give an adequate clinical and parasitological response at day 14 [ACPR_14_] > 80% [no recrudescence observed in 80% of the patients at day 14]) was predicted by simulations based on the models developed.

#### Plasmodium PI4K Sequence Analysis

Parasite DNA collected from the recrudescent *P. falciparum* population was subject to DNA sequence analysis of the PI4K gene that encodes the drug target and resistance mediator [4, 5] to investigate potential mutations that could confer resistance to MMV390048 (Supplementary Table 10).

#### Malaria Transmission to Mosquitoes

Transmission of parasites from the blood of subjects to *Anopheles stephensi* mosquitoes was assessed by a direct membrane feeding assay (DMFA) in the 40-mg cohort, and by a membrane feeding assay with serum replacement (MFA-SR) in the 80-mg cohort as described previously [12]. DMFAs and MFA-SRs were performed 12–15 days post-MMV390048 dosing on subjects who had not yet received artemether/lumefantrine treatment for recrudescence of asexual parasitemia and who had gametocytemia that made transmission possible (> 1000 gametocytes/mL). The presence of oocysts in mosquito midguts was assessed by 18S qPCR [13].

### Sample Size

The intended sample size for the SAD study (8 subjects per cohort) was calculated in accordance with the primary safety objective and to minimize the risk associated with the dose escalation from one cohort to the next. Administration of MMV390048 to 6 subjects in each dose group provided a 47%, 62%, 74%, or 82% probability of observing at least 1 occurrence of any AE with a true incidence rate for a given dose group of 10%, 15%, 20%, or 25%, respectively. Furthermore, it was assumed that pooling the data for the subjects who received placebo (2 from each cohort) would provide an adequately sized control group.

The intended sample size for the VIS (8 subjects per cohort) was based on a 2-sided *t* test at the 5% significance level, this cohort size would identify a difference of 25% in the parasite clearance rate with 80% power [7].

## RESULTS

### Study Subjects

**Figure 1.**
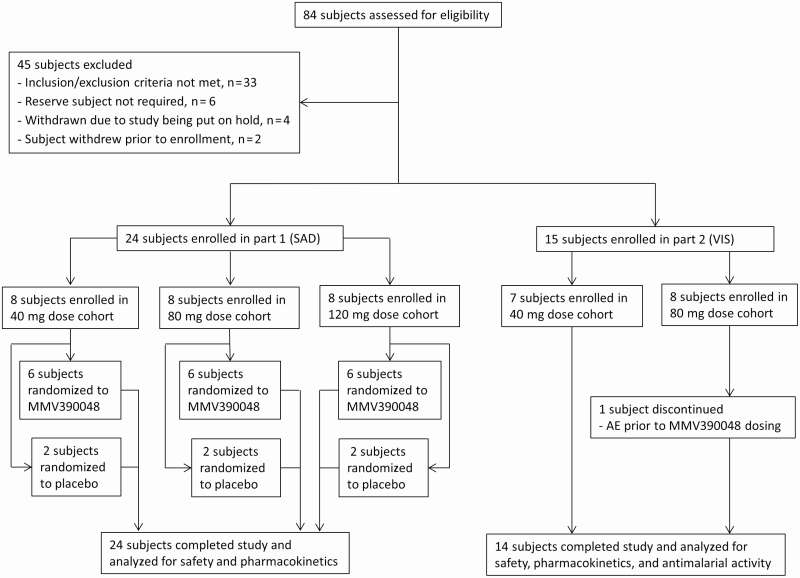
Trial profile. In part 1, SADs of MMV390048 (40–120 mg) were tested in 3 cohorts. The volunteer infection study (part 2) started after documentation of safety and pharmacokinetics data of the first cohort (40 mg dose) in part 1. Abbreviations: AE, adverse event; SAD, single ascending dose; VIS, volunteer infection study.

The study was conducted from 13 May 2016 to 16 January 2017. Subject enrollment, allocation to dose cohorts, and study completion/discontinuation are illustrated in Figure 1. Twenty-four subjects were enrolled in the SAD study (8 subjects in each cohort) and 15 in the VIS (7 subjects in the 40-mg cohort and 8 subjects in the 80-mg cohort). All subjects in both parts were male and the majority were white (Table 1). One subject was withdrawn from the VIS (80-mg cohort) before MMV390048 dosing because of an AE not related to study procedures (the subject was immediately rescued with artemether/lumefantrine). This subject was not included in PK or PD analyses.

**Table 1.** Demographic Profile of Subjects by Study and Dose Group

Characteristic	Single Ascending Dose Study				Volunteer Infection Study	
	Placebo (n = 6)	40 mg (n = 6)	80 mg (n = 6)	120 mg (n = 6)	40 mg (n = 7)	80 mg (n = 8)
Age**, y**						
Mean ± SD	33.3 ± 11.3	24.8 ± 2.5	23.0 ± 2.6	40.2 ± 13.9	30.7 ± 8.6	30.9 ± 10.0
Range	19–53	22–28	21–28	23–53	23–47	20–48
Sex, male, No. (%)	6 (100)	6 (100)	6 (100)	6 (100)	7 (100)	8 (100)
Race, No. (%)						
White	5 (83.3)	5 (83.3)	2 (33.3)	5 (83.3)	4 (57.1)	8 (100)
Asian	1 (16.7)	0 (0)	2 (33.3)	1 (16.7)	0 (0)	0 (0)
Black	0 (0)	1 (16.7)	1 (16.7)	0 (0)	0 (0)	0 (0)
Other	0 (0)	0 (0)	1 (16.7)	0 (0)	3 (42.9)	0 (0)
BMI**, kg/m**^2^						
Mean ± SD	24.1 ± 2.4	23.4 ± 1.9	24.3 ± 3.2	25.4 ± 3.0	24.3 ± 3.3	23.2 ± 4.4
Range	22.4–28.1	20.3–25.6	20.8–29.0	22.1–29.5	18.4–29.0	18.3–31.1
Height, cm						
Mean ± SD	181.3 ± 7.7	182.3 ± 9.8	173.8 ± 9.0	173.8 ± 4.4	182.9 ± 6.4	182.0 ± 7.1
Range	174.0–194.0	172.0–198.0	164.0–188.0	170.0–181.0	172.0–191.0	173.0–192.0
Weight, kg						
Mean ± SD	79.0 ± 7.1	78.1 ± 12.1	73.6 ± 12.0	76.9 ± 11.0	81.2 ± 11.4	77.3 ± 16.9
Range	67.9–85.1	64.4–95.2	58.7–95.1	66.2–92.7	58.8–92.6	56.0–101.9

Abbreviations: BMI, body mass index; SD, standard deviation.

### Safety

A total of 34 AEs were reported in 13 of the 24 subjects in the SAD study, with the majority mild in severity (n = 29/34). There was no apparent relationship between the dose of MMV390048 and the incidence or severity of AEs (Table 2 and Supplementary Table 8). The only 2 AEs considered related to MMV390048 were a mild headache in 1 subject dosed with 80 mg, and mild malaise in 1 subject dosed with 120 mg. No trends in inhibin B, follicle-stimulating hormone, luteinizing hormone, or testosterone levels were identified. There was 1 serious adverse event (SAE) reported in 1 subject dosed with placebo. Spontaneous abortion of early pregnancy occurred in the subject’s partner (the pregnancy was unknown at the time of the subject’s enrollment in the study). The SAE was not considered related to the study intervention (revealed as placebo after unblinding).

**Table 2.** Adverse Events Summary by Study and Dose Group

Adverse Event	Single Ascending Dose Study				Volunteer Infection Study	
	Placebo (n = 6)	40 mg (n = 6)	80 mg (n = 6)	120 mg (n = 6)	40 mg (n = 7)	80 mg (n = 8)
No. (%) of subjects with ≥1 adverse event						
Any AE Related to study intervention	4 (66.7) 2 (33.3)	0 (0) 0 (0)	4 (66.7) 1 (16.7)	5 (83.3) 1 (16.7)	7 (100) 2 (28.6)	8 (100) 1 (14.3)^a^
Moderate AE (grade 2)^b^ Related to study intervention	2 (33.3) 2 (33.3)	0 (0) 0 (0)	1 (16.7) 0 (0)	0 (0) 0 (0)	6 (85.7) 0 (0)	5 (62.5) 1 (14.3)^a^
Severe AE (grade 3)^b^ Related to study intervention	1 (16.7) 0 (0)	0 (0) 0 (0)	0 (0) 0 (0)	0 (0) 0 (0)	1 (14.3) 0 (0)	2 (25.0) 0 (0)
AE leading to discontinuation	0 (0)	0 (0)	0 (0)	0 (0)	0 (0)	1 (12)^c^
SAE	1 (16.7)^d^	0 (0)	(0)	(0)	0 (0)	0 (0)
No. of adverse events						
Total AEs Related to study intervention	13 7	0 0	9 1	12 1	99 3	103 1
Moderate AEs (grade 2)^b^ Related to study intervention	3 3	0 0	1 0	0 0	32 0	20 1
Severe AEs (grade 3)^b^ Related to study intervention	1 0	0 0	0 0	0 0	1 0	4 0
AEs leading to discontinuation	0	0	0	0	0	1^c^
SAEs	1^d^	0	0	0	0	0

Abbreviations: AE, adverse event; SAE, serious adverse event; PR interval, the period that extends from the beginning of the P wave until the beginning of the QRS complex.

^a^Percentage based on n = 7 for subjects who were dosed with MMV390048 in the 80-mg dose cohort.

^b^The medical assessment of AE severity was recorded in accordance with the World Health Organization Handbook for Reporting of Results of Cancer Treatment (single ascending dose study) and in accordance with the Common Terminology Criteria for Adverse Events version 4.03, 2010 (volunteer infection study).

^c^The AE leading to discontinuation was prolonged PR interval on electrocardiography, which occurred prior to MMV390048 administration.

^d^The SAE was spontaneous abortion of early pregnancy in the subject’s partner, which was not deemed related to the study intervention (placebo).

A total of 202 AEs were reported in all 15 subjects in the VIS. Most AEs were mild in severity (n = 145/202) and were considered related to malaria (n = 176/202). There was no observed difference between the 2 cohorts with respect to AE incidence or severity (Table 2 and Supplementary Table 8) and no SAEs were reported. There were 4 AEs in 3 subjects considered to be possibly related to MMV390048: mild abdominal discomfort and mild nausea in 1 subject dosed with 40 mg, moderate nausea in 1 subject dosed with 80 mg, and a possible case of mild photosensitivity in 1 subject dosed with 40 mg (very brief episode that was subjective in nature). There were 5 severe AEs recorded in 3 subjects that were all considered to be related to malaria: headache, elevated alanine aminotransferase (ALT; 519 U/L [13 times the upper limit of normal]), elevated aspartate aminotransferase (AST; 319 U/L [8 times the upper limit of normal]), and lymphopenia (n = 2, 0.41 × 10^9^/L and 0.32 × 10^9^/L). The elevations in ALT and AST occurred concomitantly in the same subject (5 days after dosing with 80 mg MMV390048), were transient and asymptomatic (Supplementary Table 9), and were not associated with elevated bilirubin. One AE resulted in a subject being withdrawn from the study on day 8 prior to MMV390048 dosing when a prolonged PR interval (peak = 219 msec) was observed on electrocardiography. The subject had a PR interval > 200 msec at both screening and before inoculation; the first-degree atrioventricular block was considered to be related to athletic conditioning causing high vagal tone.

### Pharmacokinetics

In the SAD study, absorption of MMV390048 was generally rapid with peak plasma concentrations observed at a median of 1–2 hours postdose in all groups (Table 3 and Supplementary Figure 2). Mean C_max_ and AUC_o-∞_ increased in an approximately dose-proportional manner and intersubject variability was moderate and similar across groups. MMV390048 showed a long elimination half-life, ranging from approximately 129 to 215 hours, and tended to increase with increasing dose. The PK of MMV390048 in the VIS was generally similar to the SAD study, although dose proportionality was not observed in C_max_ (Table 3 and Supplementary Figure 3)_._

**Table 3.** Pharmacokinetic Parameters by Study and Dose Group

Parameter	Single Ascending Dose Study			Volunteer Infection Study	
	40 mg (n = 6)	80 mg (n = 6)	120 mg (n = 6)	40 mg (n = 7)	80 mg (n = 7)
C_max_, ng/mL	272.7 (40.7)	561.0 (42.1)	1094.0 (36.6)	334.3 (50.0)	334.4 (77.7)
T_max_, h	1.5 (0.5–3.0)	2.0 (0.5–48.0)	1.0 (1.0–3.0)	2.0 (0.5–12.0)	2.0 (2.0–48.0)
AUC_0-last_, h × ng/mL	29 290 (65.5)	76 490 (30.0)	123 200 (28.5)	31 750 (55.1)	61 530 (40.6)
AUC_0-∞_, h × ng/mL	30 320 (66.4)	82 680 (34.3)	137 800 (33.6)	33 040 (57.0)	68 610 (45.7)
T_1/2_, h	129.4 (65.6–167.4)	161.9 (116.8–275.5)	215.2 (143.1–271.5)	102.9 (91.1–221.6)	212.8 (106.1–328.6)
CL/F, L/h	1.3 (66.4)	1.0 (34.3)	0.9 (33.6)	1.2 (57.0)	1.2 (45.7)
Vz/F, L	226.7 (35.9)	228.3 (30.9)	267.5 (18.4)	198.6 (51.5)	308.8 (41.4)

Data are geometric means (coefficient of variation), except for median (range) for T_max_ and T_1/2_.

Abbreviations: AUC_0-last_, area under the concentration-time curve from 0 hour to the last measured timepoint; AUC_0-∞_, area under the concentration-time curve from 0 hour to infinity; CL/F, apparent total clearance; C_max_, peak plasma concentration; T_1/2_, estimated elimination phase half-life; T_max_, time point at which peak plasma concentration is reached; Vz/F, apparent volume of distribution.

### Antimalarial Activity

**Figure 2.**
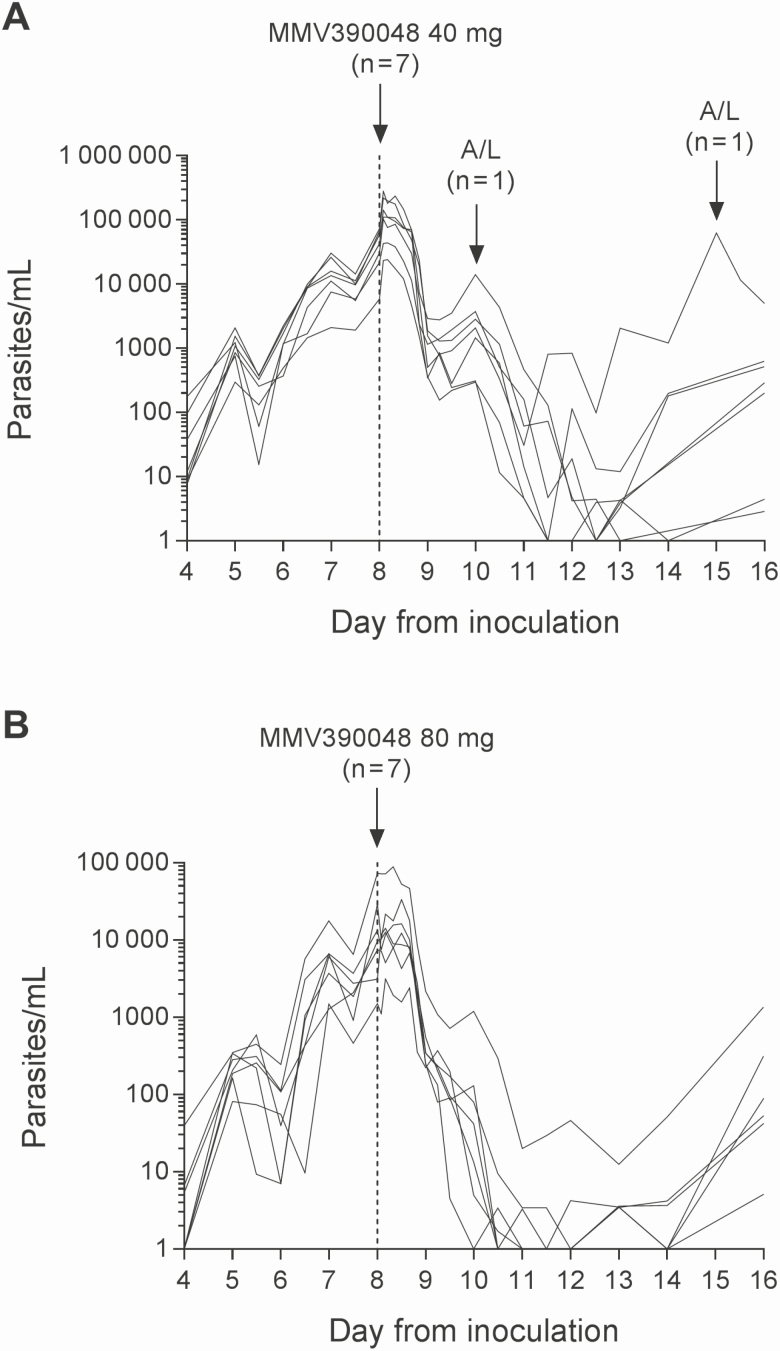
Individual subject parasitemia profiles. Subjects were inoculated intravenously with *Plasmodium falciparum*–infected erythrocytes on day 0 and were administered a single oral dose of 40-mg (*A*) or 80-mg (*B*) MMV390048 on day 8 (indicated by the vertical dashed line). Artemether/lumefantrine (A/L) was administered in response to recrudescence of parasitemia or approximately 25 days post-MMV390048 dosing if recrudescence was not observed. The parasitemia profile for 1 subject in the 80-mg dose cohort who was not dosed with MMV3900048 and was treated instead with A/L is not shown.

Initial clearance of parasitemia was observed in all subjects following MMV390048 administration in the VIS (Figure 2 and Supplementary Figures 4 and 5), although a lag of approximately 24 hours was evident. The rate of parasite clearance was relatively rapid in both cohorts (Table 4 and Supplementary Table 7), although clearance was significantly faster in the 80-mg cohort compared with the 40-mg cohort (clearance half-life, 5.5 hours [95% confidence interval {CI}, 5.2–6.0 hours] vs 6.4 hours [95% CI, 6.0–6.9 hours]; *P* = .005). Furthermore, the incidence of recrudescence was higher, and occurred earlier, in the 40-mg cohort (Table 4 and Supplementary Figures 4 and 5). Sequencing of the full-length *P. falciparum* PI4K gene on recrudescent parasites from 6 subjects in the 40-mg cohort and 3 subjects in the 80-mg cohort (Supplementary Table 11) did not reveal any mutations. All subjects were treated with artemether/lumefantrine (13 subjects also received primaquine to clear gametocytes) and were confirmed to be parasitemia free by the end of the study.

**Table 4.** Pharmacodynamics of Parasite Clearance and Incidence of Recrudescence Following MMV390048 Administration in the Volunteer Infection Study

Cohort	No.	Log_10_PRR_48_ (95% CI)	Parasite Clearance Half-life, h (95% CI)	Recrudescence, No. (%)	Day of Recrudescence Relative to Dosing
40 mg	7	2.3 (2.1–2.4)	6.4 (6.0–6.9)	6 (85.7)	2–16
80 mg	7^a^	2.6 (2.4–2.8)	5.5 (5.2–6.0)	3 (42.9)	12–19

Abbreviations: CI, confidence interval; log_10_PRR_48_, logarithm to the base 10 of the parasite reduction ratio standardized over a 48-hour period after MMV390048 administration.

^a^One subject from the 80-mg dose cohort was not dosed with MMV390048 and was excluded from analysis.

### PK/PD Modeling

A 2-compartment PK model with linear elimination, no lag time, and zero-order absorption best described the PK of MMV390048 (Supplementary Table 4). For the PD modeling, a maximum clearance rate model best described the effect of MMV390048 on the parasite growth (Supplementary Table 5). A visual predictive checkplot of the final PK/PD model is presented in Supplementary Figure 1. The PK/PD model predicted an MIC of 83 ng/mL (90% predictive interval [PI], 57–121 ng/mL), and an MPC_90_ of 238 ng/mL (90% PI, 171–330 ng/mL). Estimated key efficacy parameters of different MMV390048 doses in malaria patients are presented in Supplementary Table 6. Simulations predicted that a single dose of 120 mg MMV390048 would result in ACPR_14_ > 80% with 92% certainty, and a single dose of 140 mg would result in ACPR_14_ > 80% with 95% certainty.

### Gametocytemia and Transmission to Mosquitoes

Gametocytemia developed in all subjects following MMV390048 dosing (Supplementary Figures 4 and 5). Mosquito feeding assays were performed on a subset of subjects with transmission to mosquitoes observed in 1 of 2 subjects in the 40-mg cohort, 15 days after MMV390048 dosing, and in 3 of 4 subjects in the 80-mg cohort, 12–14 days after MMV390048 dosing (Supplementary Table 12). The highest mosquito infection rate was 10.9%, which occurred in the subject with the highest gametocytemia (5201 gametocytes/mL).

## Discussion

This study evaluated the safety, PK, and antimalarial activity of MMV390048, the first *Plasmodium* PI4K inhibitor to reach clinical development, in healthy subjects. The fact that no females were enrolled in this study due to recruitment limitations (females were eligible for inclusion in both the SAD and VIS) is a limitation, and sex differences in safety and PK properties will need to be investigated in future studies.

The safety profile of MMV390048 in this study is consistent with that observed in the 3 previous studies where MMV390048 was administered to 64 healthy subjects [6], confirming safety and tolerability up to a single oral dose of 120 mg. A case of possible photosensitivity was reported by 1 subject in the current study 4 days after dosing with 40 mg MMV390048 in the VIS. However, the event was very brief (approximately 30 seconds) and subjective in nature. Photosensitivity was not reported in any of the 3 previous studies. However, as mild phototoxicity was observed in preclinical toxicology studies, phototoxicity should be carefully monitored in further clinical development. Transient severe elevations in ALT and AST were observed in 1 subject in the VIS. Such changes have been previously reported in experimental malaria infection studies [14] as well as in natural infection [15], and are considered a consequence of the inflammatory process associated with malaria infection.

A single dose of 40 mg or 80 mg MMV390048 resulted in initial parasite clearance in subjects with low-level *P. falciparum* infection (1000–100 000 parasites/mL). The observed lag of approximately 24 hours between dosing and parasite clearance is consistent with the in vitro antimalarial activity of MMV390048 [4]. The rate of initial parasite clearance was similar to that recorded for other antimalarials administered as a single dose in the IBSM model; 40 mg MMV390048 clears parasites at a rate comparable to 200 mg artefenomel [16], 800 mg ferroquine [14], and 10 mg/kg mefloquine [17], whereas 80 mg MMV390048 clears parasites at a rate comparable to 640 mg piperaquine [9]. As expected, recrudescence occurred in several subjects (6 subjects in the 40-mg cohort and 3 subjects in the 80-mg cohort) after initial parasite clearance. Genetic mutations conferring resistance to MMV390048 were not observed in any recrudescent infections at loci where it could be induced in vitro [4]. Due to the small sample size tested, emergence of MMV390048 resistance needs to continue being monitored during clinical development.

Simulations based on PK/PD modeling predicted that a single dose of 120 mg MMV390048 would likely achieve cure in patients with *P. falciparum* malaria (ACPR_14_ >80% with 92% certainty). This is higher than preclinical prediction (80–100 mg) [4]. These simulations have informed dose selection for a phase 2a study under way in Ethiopia where single doses of MMV390048 are being tested in adult patients with acute, uncomplicated *P. falciparum* or *Plasmodium vivax* malaria monoinfection (ClinicalTrials.gov NCT02880241). We have previously estimated the single efficacious dose of candidate antimalarials including DSM265 [18] and ferroquine [14] using the IBSM model. The dose estimate for DSM265 was subsequently shown to be accurate in patients with uncomplicated *P. falciparum* malaria [19]. This model is therefore showing the potential to expedite the development of new antimalarials, which is urgently needed to combat drug resistance [20].

Gametocytemia was detected in all subjects 1–2 weeks after administration of MMV390048, and transmission to mosquitoes occurred at levels similar to that observed after treatment with low-dose piperaquine [12]. This indicates that a single dose of 40 or 80 mg MMV390048 does not abrogate the potential for transmission. However, MMV390048 may affect late-stage gametocyte viability and reduce transmission, as demonstrated in preclinical studies [4], but this was not evaluated here. The selection of a partner drug with MMV390048 to block malaria transmission should be considered in any combinations.

In conclusion, this study successfully characterized the antimalarial activity of a single dose of MMV390048 in experimental blood-stage *P. falciparum* infection. The pharmacokinetics and pharmacodynamics of the compound support its development as a partner drug of a new, potentially single-dose combination therapy for clinical malaria.

## Supplementary Data

Supplementary materials are available at *Clinical Infectious Diseases* online. Consisting of data provided by the authors to benefit the reader, the posted materials are not copyedited and are the sole responsibility of the authors, so questions or comments should be addressed to the corresponding author.

ciaa368_suppl_Supplementary_Methods

## Notes

***Acknowledgments*.** The authors thank all of the volunteers who participated in the study; staff at Q-Pharm who conducted the trial; staff at the University of Cape Town, Division of Clinical Pharmacology Laboratory for plasma drug concentration measurements; and staff at the Queensland Paediatric Infectious Diseases laboratory for quantitative polymerase chain reaction analysis. From the QIMR Berghofer Medical Research Institute, the authors thank Lachlan Webb for assisting with the pharmacodynamic analysis, Dr Rebecca Watts for project management, Hayley Mitchell for assistance with mosquito rearing and transmission studies, and Dr Adam Potter for manuscript preparation.

***Disclaimer.*** The donors had no role in the design, conduct, or analysis of the trial.

***Financial support*.** This work was funded by Medicines for Malaria Venture (MMV). MMV is funded by a number of donors, including the United States Agency for International Development, the Bill & Melinda Gates Foundation, the United Kingdom Department for International Development, the Norwegian Agency for Development Cooperation, Irish Aid, Newcrest Mining Ltd, Australian Aid, the Swiss Agency for Development and Co-operation, and the Wellcome Trust. Funding was also received from the National Institutes of Health (grant number R01 AI109023 to D. A. F.). J. S. M. was supported by a National Health and Medical Research Council Practitioner Fellowship.

***Potential conflicts of interest*.** J. J. M., N. G., C. D., M. H. C., and S. C. are employed by MMV, the funder of the study. J. S. M., as the principal investigator, received funding from MMV to perform the study. All other authors report no potential conflicts. All authors have submitted the ICMJE Form for Disclosure of Potential Conflicts of Interest. Conflicts that the editors consider relevant to the content of the manuscript have been disclosed.
