# Supplementary material for: A Phase 1, Placebo-controlled, Randomized, Single Ascending Dose Study and a Volunteer Infection Study to Characterize the Safety, Pharmacokinetics, and Antimalarial Activity of the Plasmodium Phosphatidylinositol 4-Kinase Inhibitor MMV390048
Source: Clin Infect Dis. 2020 Apr 2;71(10):e657–64. doi: 10.1093/cid/ciaa368 (PMC7744986; doi:10.1093/cid/ciaa368)
Supplement: ciaa368_suppl_Supplementary_Methods [file ciaa368_suppl_supplementary_methods.pdf]

Supplementary appendix

A phase 1, placebo controlled, randomised, single ascending dose study and a volunteer infection study to characterize the safety, pharmacokinetics and antimalarial activity of the *Plasmodium* phosphatidylinositol 4-kinase inhibitor MMV390048

James S. McCarthy, Cristina Donini, Stephan Chalon, John Woodford, Louise Marquart, Katharine A. Collins, Felix D. Rozenberg, David A. Fidock, Mohammed H. Cherkaoui-Rbati, Nathalie Gobeau, Jörg J. Möhrle

Contents

Subject inclusion and exclusion criteria 3

Pharmacokinetic/pharmacodynamic (PK/PD) modelling methods and results 8

Table S1. Studies included in PK and PD datasets 9

Table S2. Summary of gender and race within the PK and PD datasets 9

Table S3. Summary of age, BMI, body weight and height within the PK and PD datasets 9

Table S4. Population parameter estimates of the final covariate PK model 10

Table S5. Population estimates of the final PD model 11

Table S6. Population and range value of MIC, time above MIC, MPC_{90}, time above MPC_{90}, maximum PRR_{48} and total PRR 11

Figure S1. Model validation representative visual predictive check from final pharmacodynamic model of MMV390048: Parasitemia (log scale) vs. actual time relative to dosing stratify by dose level. 12

Individual subject MMV390048 plasma concentration-time profiles 13

Figure S2. Individual subject MMV390048 plasma concentration/time profiles (single ascending dose study). 13

Figure S3. MMV390048 concentration-time profiles by dose cohort (volunteer infection study) .. 14

Individual subject parasitemia and gametocytemia profiles 15

Figure S4. Individual subject parasitemia and gametocytemia profiles following dosing with 40 mg MMV390048 15

Figure S5. Individual subject parasitemia and gametocytemia profiles following dosing with 80 mg MMV390048 16

Individual subject parasite clearance rates following MMV390048 treatment 17

Table S7. Individual $log_{10}PRR_{48}$ and parasite clearance half-life 17

Safety results 18

Table S8. Summary of adverse events by dose cohort 18

Table S9. Alanine transaminase and aspartate transaminase values recorded during the study for the subject presenting with severe elevations 21

MMV390048 resistance assessment methods and results 22

Table S10. *S*equencing primers and protocols for each nested PCR reaction 22

Table S11. *Plasmodium* PI4K gene sequencing 23

Malaria transmission results 24

Table S12. Membrane feeding assays 24

References 25

Subject inclusion and exclusion criteria

Inclusion Criteria

Subjects eligible for inclusion in this study must have fulfilled all of the following criteria:

1. Completion of the written informed consent process.

2. Men or WNCBP age 18 to 55 years, in good health as determined by past medical history, physical examination, vital signs, electrocardiogram, and laboratory tests at screening.

3. Male subjects agree to use acceptable methods of contraception if the male subject's partner could become pregnant from the time of the first administration of study medication until 130 (90+40) days following administration of the investigational medicinal product. One of the following acceptable methods of contraception must be utilized:

a. Condom and occlusive cap (diaphragm or cervical/vault caps)

b. Surgical sterilization (vasectomy with documentation of azoospermia) and a barrier method (condom or occlusive cap [diaphragm or cervical/vault caps].

c. The subject's female partner uses oral contraceptives (combination estrogen/progesterone pills), injectable progesterone or subdermal implants (commenced at least 14 days prior to IMP administration to the male subject) and a barrier method (condom or occlusive cap [diaphragm or cervical/vault caps].

d. The subject's female partner uses a medically prescribed topically applied transdermal contraceptive patch (commenced at least 14 days prior to IMP administration to the male subject) and a barrier method (condom or occlusive cap [diaphragm or cervical/vault caps].

e. The subject's female partner has undergone documented tubal ligation (female sterilization). In addition, a barrier method (condom or occlusive cap [diaphragm or cervical/vault caps].

f. The subject's female partner has undergone documented placement of an intrauterine device or intrauterine system. In addition, a barrier method (condom or occlusive cap [diaphragm or cervical/vault caps].

g. True abstinence: when this is in line with the preferred and usual lifestyle of the subject. Periodic abstinence (e.g. calendar, ovulation, symptothermal, post-ovulation methods) and withdrawal are not acceptable methods of contraception. Abstinent subjects (in consultation with their partners) have to agree to use 1 of the above-mentioned contraceptive methods, if they start sexual relationships during the study and for up to 100 days after the last dose of study drug.

4. Women subjects must be of non-childbearing potential (WNCBP) as per one of the following definitions:

a. Natural (spontaneous) post-menopausal defined as being amenorrhoeic for at least 12 months without an alternative medical cause with a screening follicle stimulating hormone level consistent with local laboratory levels for post-menopause.

b. Premenopausal with irreversible surgical sterilization by hysterectomy and/or bilateral oophorectomy or salpingectomy at least 6 months before screening (as determined by subject medical history).

5. Haematology, clinical chemistry and urinalysis results at screening that are within the local laboratory reference range or, if outside the range, not clinically significant as judged by the Investigator in accordance with Sponsor-approved clinically acceptable laboratory ranges, documented prior to study start. More specifically, serum creatinine, hepatic transaminase enzymes (AST ALT), and total bilirubin (unless the subject

has documented Gilbert syndrome) should not exceed the ranges approved by the Sponsor as acceptable, and haemoglobin must be equal to or higher than the lower limit of the normal range.

6. Total body weight greater than 50 kg and a body mass index (BMI) within the range of 18 to 32 kg/m^2 (inclusive).

7. Non-smoker or ex-smoker for more than 90 days prior to screening, or smoke no more than 5 cigarettes per day as determined by history. Must be able to abstain from smoking during the inpatient stay.

8. Willing and able to comply with all scheduled visits, treatment plan, laboratory tests, and other study procedures.

9. Agree to stay in contact with the study site for the duration of the study and up to 2 weeks following the end of study visit, provide updated contact information as necessary, and have no current plans to move away from the study area for the duration of the study.

Exclusion Criteria

Subjects fulfilling any of the following criteria were not eligible for inclusion in this study:

1. Male subjects with a female partner(s) who is (are) pregnant or lactating from the time of the administration of study medication.

2. Women of childbearing potential, defined as all women physiologically capable of becoming pregnant, including women whose career, lifestyle, or sexual orientation precludes intercourse with a male partner and women whose partners have been sterilized by vasectomy or other means.

3. Evidence or history of clinically significant haematological, renal, endocrine, pulmonary, gastrointestinal (including gallbladder), cardiovascular (including a family history of long QT syndrome or sudden death), hepatic, psychiatric, neurologic, or allergic disease (including drug or food allergies, anaphylaxis or other severe allergic reactions but excluding untreated, asymptomatic, seasonal allergies at the time of dosing).

4. History of malignancy of any organ system (other than localised basal cell carcinoma of the skin), treated or untreated, within the past five years, regardless of whether there is evidence of local recurrence or metastases.

5. Other severe acute or chronic medical or psychiatric condition or laboratory abnormality that may increase the risk associated with study participation or investigational product administration or may interfere with the interpretation of study results and, in the judgment of the Investigator, would make the subject inappropriate for entry into this study.

6. Any surgical or medical condition possibly affecting drug absorption (e.g. cholecystectomy, gastrectomy, bowel disease, etc.), distribution, metabolism or excretion.

7. Previous splenectomy.

8. A history of photosensitivity.

9. Subject positive for any of the following:

- o Anti-human immunodeficiency virus 1 or 2 antibodies (anti-HIV1 or anti-HIV2 Ab) (ELISA)
- o Hepatitis B surface antigen (HBsAg)
- o Anti-hepatitis B core antibodies (anti-HBcAb)

o Anti-hepatitis C antibodies (anti-HCV)

10. Resting vital signs (measured after 5 minutes in the supine position) at screening, pre-dose (Part A) or pre-inoculation (Part B) outside of the following study-specific normal ranges:

o body temperature < 38.0 °C (tympanic for Part A; sublingual for Part B)

o 90 < SBP < 140 mmHg

o 50 < DBP < 90 mmHg

o 40 < pulse rate < 100 bpm

11. Symptomatic postural hypotension at screening, irrespective of the decrease in blood pressure, or asymptomatic postural hypotension defined as a decrease in systolic blood pressure ≥20 mmHg 2 minutes after changing from a supine to standing position.

12. A history of clinically significant ECG abnormalities, or any of the following ECG abnormalities at screening, pre-dose (Part A and B) or pre-inoculation (Part B):

o PR >210 ms

o QRS complex >120 ms

o QTcF >450 ms

o Second or third degree atrioventricular block

o Incomplete, complete or intermittent bundle branch block

o Abnormal T wave morphology

o Left ventricular hypertrophy with repolarisation abnormalities

o Right ventricular hypertrophy.

13. Presence of acute infectious disease or fever (i.e. body temperature ≥38.5 °C) within five days prior to the first dose of study medication (Part A, tympanic measurement) or the inoculation administration (Part B, sublingual measurement).

14. Use of prescription or non-prescription drugs, herbal and dietary supplements within 14 days or 5 half-lives (whichever is the longer) prior to the first dose of study medication (Part A) or the inoculation administration (Part B). [As an exception, ibuprofen (preferred) may be used at doses of up to 1.2 g/day, or paracetamol at doses of up to 1 g/day (Part A) or 2 g/day (Part B). Limited use of other non-prescription medications not believed to affect subject safety or the overall results of the study, may be permitted on a case-by-case basis following approval by the Sponsor in consultation with the Investigator.]

15. Recipient of any vaccination within 28 days prior to the first dose of study medication (Part A) or the inoculation administration (Part B).

16. Urine drug screen at screening, pre-dose (Part A) or pre-inoculation (Part B) positive for any drug as listed in Section 9.2.4 unless there is an explanation acceptable to the medical Investigator (e.g. the subject has stated in advance that they consumed a prescription or over the counter product which contained the detected drug) and/or the subject has a negative urine drug screen on retest by the pathology laboratory.

17. Ingestion of any poppy seeds within the 24 hours prior to the screening blood test.

18. A positive alcohol breath test at screening, pre-dose (Part A) or pre-inoculation (Part B).

19. History of regular alcohol consumption exceeding a weekly intake of more than 21 units for males and more than 14 units for females (one unit is equivalent to 8-10 g of ethanol, 285 ml of beer or lager, one glass [125 ml] of wine, or 25 ml of spirits) within 6 months of screening.

20. History of drug habituation, or any prior intravenous usage of an illicit substance.

21. Participation in any investigational product study within 12 weeks or five half-lives (whichever is longer) prior to the first dose of the study medication.

22. Intake of grapefruit, grapefruit juice or grapefruit-related citrus fruits (e.g. Seville oranges, pomelos) within 28 days prior to the first dose of the study medication.

23. Excessive consumption of beverages containing xanthine bases (e.g. more than 400 mg of caffeine per day, equivalent to approximately 4 cups of coffee).

24. Pregnant or nursing (lactating) women.

25. Participation in any research study involving blood sampling (more than 450 ml/ unit of blood), or blood donation to the Australian Red Cross Blood Service (ARCBS) or other blood bank during the 8 weeks prior to IMP administration (Part A) or inoculation (Part B).

26. Blood donation (excluding plasma donation) of any volume, within 1 month prior to screening.

27. Medical requirement for intravenous immunoglobulin or blood transfusions.

28. Subject with poor peripheral venous access.

29. Subject unwilling or unable to comply with the restrictions described in this protocol.

30. Any subject who, in the judgment of the Investigator, is likely to be noncompliant during the study, or unable to cooperate because of a language problem or poor mental development.

31. Any subject who is the Investigator or any sub-investigator, research assistant, pharmacist, study coordinator, or other staff thereof, directly involved in conducting the study.

32. Recent (within the last three years) and/or recurrent history of autonomic dysfunction (e.g. recurrent episodes of fainting, palpitations, etc.).

Additionally for subjects in Part B of the study only:

33. Any history of malaria.

34. Participation in a previous malaria challenge study or Part A of the current study.

35. Participation in a malaria vaccine trial.

36. Has travelled to or lived (for more than 2 weeks) in a malaria-endemic area during the past 12 months.

37. Any plan to travel to a malaria-endemic country during the course of the study.

38. Evidence of increased cardiovascular disease risk (defined as >10%, 5 year risk for those greater than 35 years of age, as determined by the Australian Absolute Cardiovascular Disease Risk Calculator. Risk factors include sex, age, systolic blood pressure (mm/Hg), smoking status, total and HDL cholesterol (mmol/L), and reported diabetes status.

39. Frequent headaches and/or migraine, recurrent nausea, and/or vomiting (more than twice a month).

40. Subject unwilling to defer blood donations to the ARCBS for 6 months.

41. Subject who has ever received a blood transfusion.

42. Subject currently receiving, or having previously received, immunosuppressive therapy (including systemic steroids, adrenocorticotrophic hormone or inhaled steroids) at a dose or duration associated with hypothalamic-pituitary-adrenal axis suppression (e.g. 1 mg/kg/day of prednisone or its equivalent, or chronic use of inhaled high potency corticosteroids such as budesonide 800 μg per day or fluticasone 750 μg).

43. Any recent (<6 weeks) or current systemic therapy with drugs known to have potential antimalarial activity listed in Section 6.5.2 of the study protocol.

44. Known allergy to one of the antimalarial rescue medications proposed for the challenge study.

45. Subject is unwilling to abstain from consumption of quinine containing foods/beverages such as tonic water, lemon bitter, from inoculation (Day In0) to the end of the antimalarial treatment.

46. Subject lives alone (at any stage from Day In0 until at least the end of the antimalarial drug treatment).

Pharmacokinetic/pharmacodynamic (PK/PD) modelling methods and results

Objectives

(1) Evaluation of the population pharmacokinetics of MMV390048 including potential covariates.

(2) Characterization of the PK/PD relation of MMV390048 for killing *P. falciparum* parasites, and calculation of the derived pharmacological parameters: minimum inhibitory concentration (MIC), minimum parasiticidal concentration that achieves 90% of the maximum effect (MPC_{90}) and parasite reduction ratio over 48 hours (PRR_{48}).

(3) Prediction of the efficacious dose needed to clear 10^9 parasites/mL.

(4) Prediction of the efficacious dose for a phase IIa study, such as it leads to an adequate clinical and parasitological response at day 14 ($ACPR_{14}$) larger than 80% with 95% certainty.

(5) Estimation of the MIC, the time above MIC the MPC_{90}, the time above MPC_{90}, the PRR_{48} and total parasite reduction ratio (PRR_{total}) of all predicted doses for patients.

Methods

All data processing, analysis, model setup and modelling result analysis were conducted within R (Microsoft Open R v3·4·3) combined to the IQR package (v0·7·2) and Monolix (2016R1). First, a population PK model was built with the PK data of the single ascending dose, formulation optimisation, and malaria challenge studies (Table S1). Then a population PK/PD model was developed with data from the malaria challenge studies (Table S1), where the individual PK parameters estimated during the initial stage of PK modelling were used as regression parameters, and only the PD parameters were estimated. The demographic characteristics of subjects included in the PK and PD datasets are summarised in Tables S2 and S3.

Simulations were undertaken in order to predict the efficacious dose defined as the dose that clears 10^9 parasites/mL in a large population. For this purpose, 100 trials composed each of 100 men, which have been found to have a faster clearance than women, thus need a higher dose, were simulated for various doses. The selected formulation was fixed to formulation A (MMV390048 tartaric acid formulation). The parasite growth rate was assumed to be 10-fold per 48 hours' lifecycle (0·048 hr^{-1}). The efficacious dose was calculated as the dose with a median PRR_{total} of 10^9.

Further simulations were performed to help decide the dose to be tested in the first cohort of a planned phase IIa clinical study. The baseline parasitemia was randomly drawn for each patient from a previous phase IIa study in Peru (Median: $10^{6·86}$ parasites/mL - Range: $10^{5·76}$;$10^{7·85}$) [1]. Formulation A was also selected, and the same assumption was made for the parasite growth rate as the previous simulations. The parasitemia profiles of 200 trials of 17 subjects (the planned size of the first cohort, and assuming 93% of men) were simulated up to day 14. For each trial, the $ACPR_{14}$ was calculated as the proportion of patients that were malaria-free, i.e. if parasitemia was below the lower limit of quantitation (LLOQ) of 10 parasites/mL at day 14, or if the parasitemia was below a cure threshold of 1 parasite within 5 L of blood at any time before day 14. From those simulations, the predicted dose was the dose for which the $5th$ quantile of the $ACPR_{14}$ was equal to 80%. This corresponds to the success of the first cohort, defined as having an $ACPR_{14}$ larger than 80% with 95% certainty.

Table S1. Studies included in PK and PD datasets

Study name (ClinicalTrials.gov identifier)	MMV390048 dose	No. of subjects
First in human study, powder-in-bottle formulation (NCT02230579)	0 mg	12
	5 mg	6
	20 mg	6
	40 mg	6
	40 mg (Fed)	6
	80 mg	6
	120 mg	6
Formulation optimisation study, tablet formulation (NCT02554799)	40 mg	18
Single ascending dose study, tablet formulation (NCT02783820)	0 mg	6
	40 mg	6
	80 mg	6
	120 mg	6
Malaria challenge study, powder-in-bottle formulation (NCT02281344)	20 mg	6
Malaria challenge study, tablet formulation (NCT02783833)	0 mg	1
	40 mg	7
	80 mg	7
Total		111

Table S2. Summary of gender and race within the PK and PD datasets

Characteristic	Category	PK dataset (N=111)	PD dataset (N=21)
Gender	Female	8 (7·2%)	0 (0%)
	Male	103 (92·8%)	21 (100%)
Race	Asian	5 (4·5%)	0 (0%)
	Black	35 (31·5%)	0 (0%)
	Black African	2 (1·8%)	0 (0%)
	Black Or African American	2 (1·8%)	0 (0%)
	Caucasian	13 (11·7%)	0 (0%)
	Mixed Race/Cape Colored	11 (9·9%)	0 (0%)
	Other	8 (7·2%)	3 (14·3%)
	White	35 (31·5%)	18 (85·7%)

N: Number of subjects. Number of subjects in each category and percentage within this category.

Table S3. Summary of age, BMI, body weight and height within the PK and PD datasets

Characteristic	PK dataset (N=111)	PD dataset (N=21)
Age (years)	30·9 (10·1) [19-54]	29·2 (8·2) [19-48]
BMI (kg/m^2)	24 (3·3) [18·2-31·4]	23·9 (4.0) [18·3-31·1]
Body Weight (kg)	74·2 (12·8) [52·8-108]	79·8 (14·6) [56-108]
Height (cm)	176 (8·5) [156-198]	182 (6·6) [170-192]

N: Number of subjects. Entries represent: Mean (Standard deviation) [Minimum-Maximum].

Results

A two-compartment PK model with linear elimination, no lag time and zero-order absorption was developed to describe the PK of MMV390048 after single doses up to 120 mg. Furthermore, gender, formulation and fasted state were used as covariates to describe the clearance CL, the bioavailability $F_{abs,0}$ and the absorption time $T_{k,0}$, respectively (Table S4).

For the PD modelling, an E_{max}-model was used to describe the effect of MMV390048 on the parasite growth. This model assumes an exponential growth of the parasites and a direct effect of concentrations on parasite killing/clearance rate, such as in log-scale:

$$\begin{cases} \dfrac{dPL}{dt} & = & GR - E_{max} \cdot \dfrac{C_c^{Hill}}{C_c^{Hill} + EC_{50}^{Hill}} \\ PL(t_0) & = & PL_{base} \end{cases}$$

where PL_{base} is the initial parasitemia at time t_0 (time of first observation), C_c the concentration in the central compartment, E_{max} the maximum effect of the drug, EC_{50} the concentration that results in 50% of the maximum effect and $Hill$ is the hill coefficient. Table S5 summarises the estimates of the final PD model. A model validation representative visual predictive check from the final pharmacodynamic model of MMV390048 is presented in Figure S1.

Then, simulations predicted that a dose of 55 mg is sufficient to clear 10^9 parasites/mL. Furthermore, the dose that leads to an $ACPR_{14}$ larger than 80% with 95% certainty within a cohort of 17 patients was predicted to be 140 mg, which is higher than the maximum tested dose of 120 mg. Nevertheless, a dose of 120 mg, according to the simulations, would lead to an $ACPR_{14}$ larger than 80% with 92% certainty, which might be enough to guarantee success.

Finally, Table S6 provides the estimated key parameters MIC, time above MIC, MPC_{90}, time above MPC_{90}, PRR_{48}, and PRR_{total} for patients for different doses. To be noted that the value of MIC and PRR_{48} are different than those estimated from the population PD model as the growth rate used for the simulations is different than the growth rate estimated in the human challenge.

Table S4. Population parameter estimates of the final covariate PK model

PARAMETER	VALUE	RSE	SHRINKAGE	COMMENT
$F_{abs,0}$	1 (FIX)	-	-	Relative bioavailability (-)
CL	0·616	20·6%	-	Apparent clearance (L/hour)
V_c	138	9·71%	-	Apparent central volume (L)
Q_1	30·2	13·7%	-	Apparent intercompartmental clearance (L/hour)
$V_{p,1}$	115	8·84%	-	Apparent peripheral volume (L)
$T_{k,0}$	1·43	7·55%	-	Absorption time (hours)
$T_{lag,1}$	0 (FIX)	-	-	Absorption lag time (hours)
**Inter-individual variability**				
$\omega_{F_{abs,0}}$	0·426	9·5%	12%	Log-Normal
ω_{CL}	0·455	8·95%	13%	Log-Normal
ω_{V_c}	0·457	14·2%	27%	Log-Normal
ω_{Q_1}	0 (FIX)	-	-	Log-Normal
$\omega_{V_{p,1}}$	0 (FIX)	-	-	Log-Normal
$\omega_{T_{k,0}}$	0·617	8·07%	14%	Log-Normal
$\omega_{T_{lag,1}}$	0 (FIX)	-	-	Normal
**Parameter-Covariate relationships**				
$\beta_{F_{abs,0},FORM_2}$	0·449	39·2%	-	MMV390048 Formulation B on Fabs0
$\beta_{F_{abs,0},FORM_3}$	-0·471	22·4%	-	MMV390048 Formulation PIB on Fabs0
β_{CL,SEX_2}	0·555	35·9%	-	Gender Male on CL
$\beta_{T_{k,0},FOOD_2}$	1·09	24·6%	-	MMV390048 Food Status Fed on Tk0
**Residual Variability**				
$error_{ADD1}$	3e-04	19·2%	-	Compound concentration (μg/mL)
$error_{PROP1}$	0·213	2·2%	-	Compound concentration (μg/mL)
Objective function	-7683	-	-	-

Table S5. Population estimates of the final PD model

PARAMETER	VALUE	RSE	SHRINKAGE	COMMENT
PL_{base}	-3·05	13·2%	-	Log-transformed baseline parasitemia (percent or 1/mL)
PL_{err}	0 (FIX)	-	-	Individual deviation from baseline parasitemia
GR	0·0674	4·13%	-	Net parasite growth rate (1/hour)
E_{max}	0·243	9·16%	-	Maximum clearance rate (1/hour)
EC_{50}	0·114	7·89%	-	Concentration achieving 50 percent of maximum effect (µg/mL)
$Hill$	3 (FIX)	-	-	Hill coefficient (.)
**Inter-individual variability**				
$\omega_{PL_{base}}$	0·2 (FIX)	-	-	Normal
$\omega_{PL_{err}}$	0 (FIX)	-	-	Normal
ω_{GR}	0·0869	17%	4.9%	Log-Normal
$\omega_{E_{max}}$	0·2 (FIX)	-	-	Log-Normal
$\omega_{EC_{50}}$	0·2 (FIX)	-	-	Log-Normal
ω_{Hill}	0 (FIX)	-	-	Normal
**Residual Variability**				
$error_{ADD1}$	1·19	3·11%	-	Log-transformed parasitemia (percent or 1/mL)
Objective function	1489	-	-	-

Table S6. Population and range value of MIC, time above MIC, MPC_{90}, time above MPC_{90}, maximum PRR_{48} and total PRR.

Dose	MIC (ng/mL)	Time Above MIC (days)	MPC_{90} (ng/mL)	Time Above MPC_{90} (days)	$PRR_{48,max}$ ($\log_{10}$)	PRR_{tot} ($\log_{10}$)
55 mg	71·7 [63·1;80·4]	10·9 [7·9;17·4]	237·2 [208;266·4]	0·2 [0·1;1]	4·1 [3·3;4·8]	9·8 [6·3;16·4]
120 mg	-	18·6 [14·2;27·4]	-	6·7 [4·5;10·5]	-	24·3 [17·5;37]
140 mg	-	20·3 [15·3;28]	-	8·4 [5·9;12·9]	-	27·6 [19·3;41·5]

Values between brackets represent the 90% confidence interval.

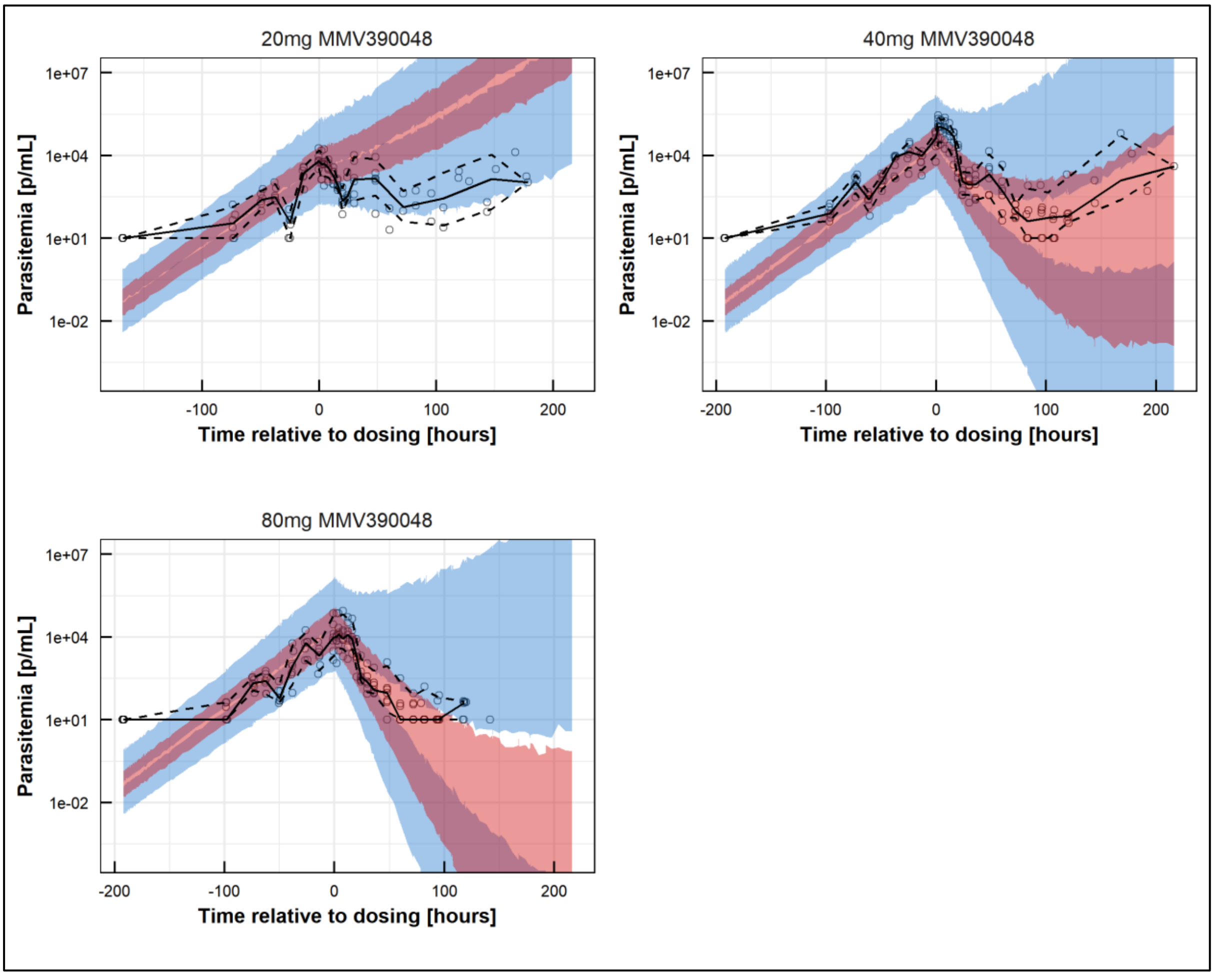

Figure S1. Model validation representative visual predictive check from final pharmacodynamic model of MMV390048: Parasitemia (log scale) vs. actual time relative to dosing stratified by dose level.

Dots are observed data and the solid lines represent the 5th, 50th, and 95th percentiles of observed data, respectively. The shaded areas represent the simulation-based 90% confidence intervals for the 5th, 50th, and 95th percentiles of the predicted data (500 simulations).

Individual subject MMV390048 plasma concentration-time profiles

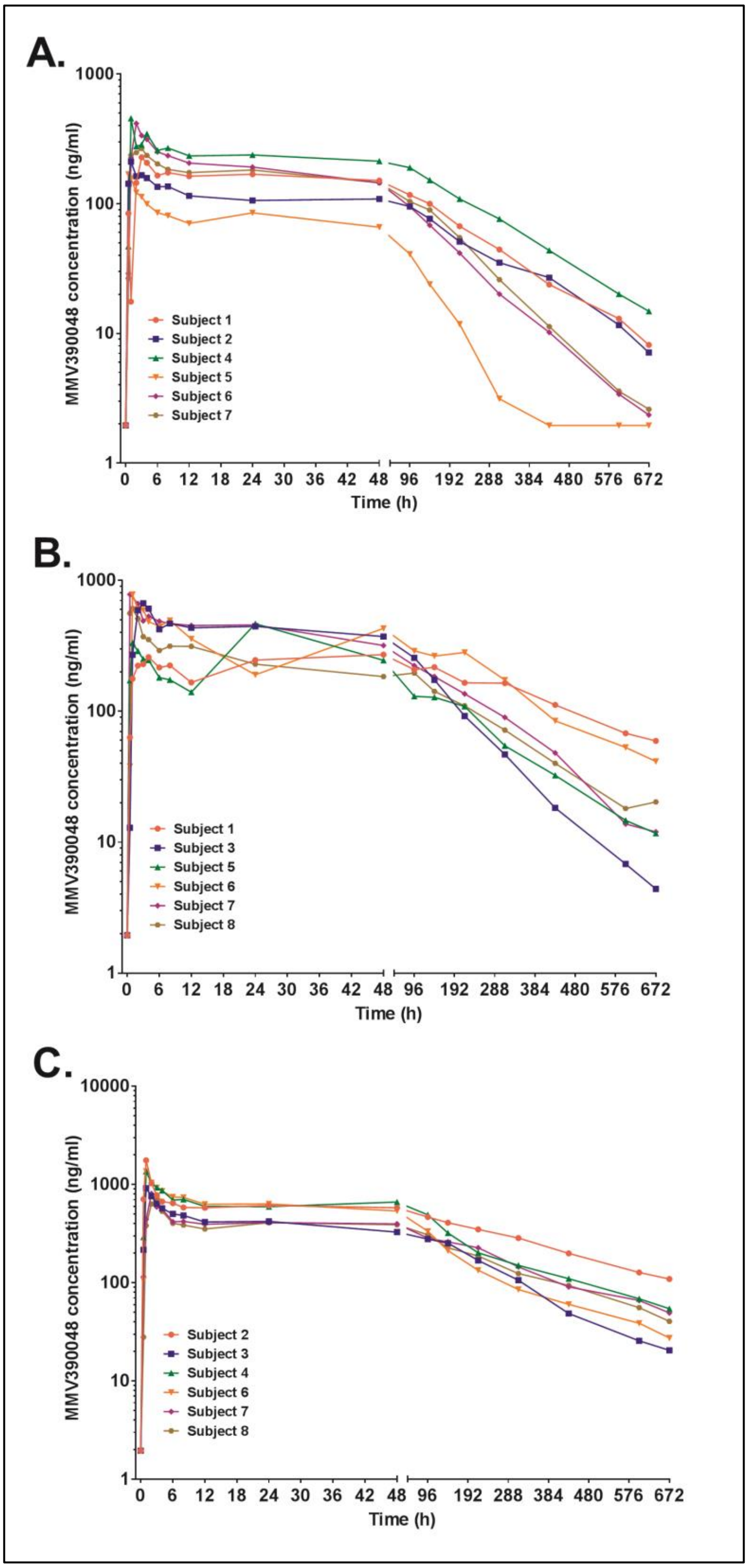

Figure S2. Individual subject MMV390048 plasma concentration/time profiles (single ascending dose study). Individual profiles of subjects in the 40 mg (A), 80 mg (B), and 120 mg (C) dose cohorts. Subjects dosed with placebo are not included in the graph.

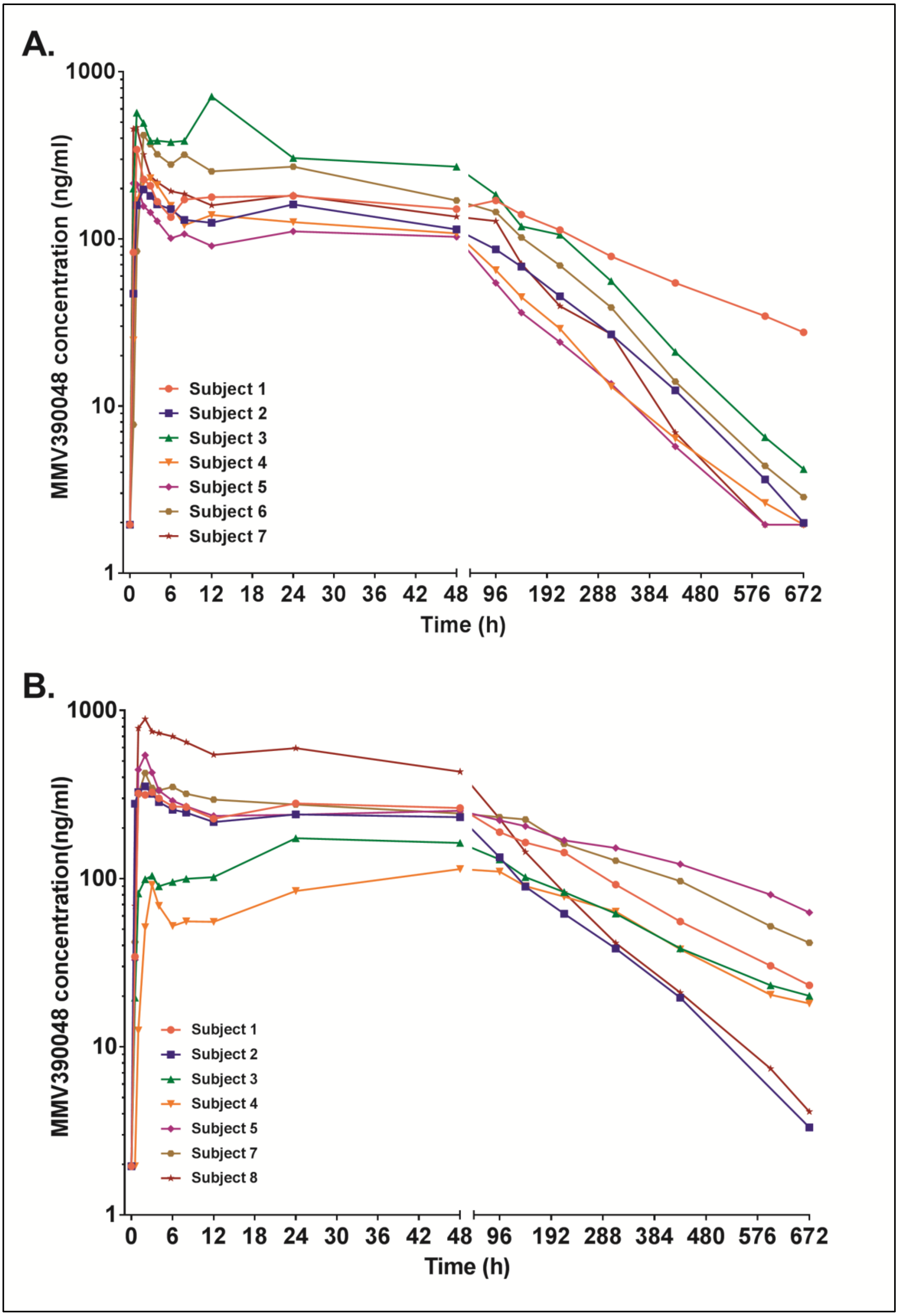

Figure S3. MMV390048 concentration-time profiles by dose cohort (volunteer infection study). Individual profiles of subjects in the 40 mg (A) and 80 mg (B) dose cohorts. Subject 6 in the 80-mg dose cohort was not dosed with MMV390048 and therefore is not included in the graph.

Individual subject parasitemia and gametocytemia profiles

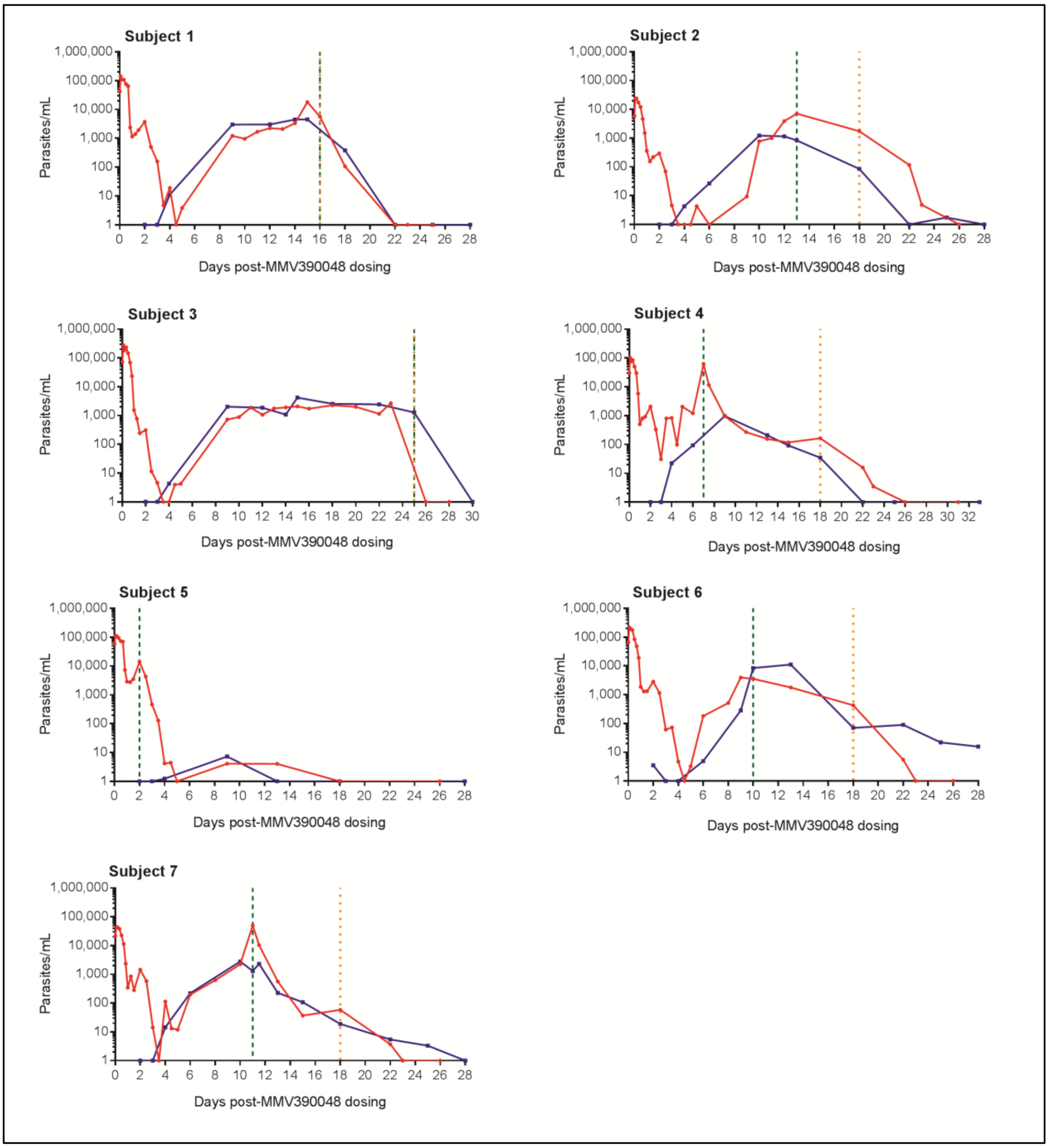

Figure S4. Individual subject parasitemia and gametocytemia profiles following dosing with 40 mg MMV390048. Total parasitemia measured by quantitative PCR targeting the gene encoding 18S rRNA is represented in red. Gametocytemia measured by quantitative reverse transcriptase PCR targeting *pfs25* mRNA is represented in blue. Green dashed lines represent time of treatment with artemether/lumefantrine. Orange dashed lines represent time of treatment with primaquine.

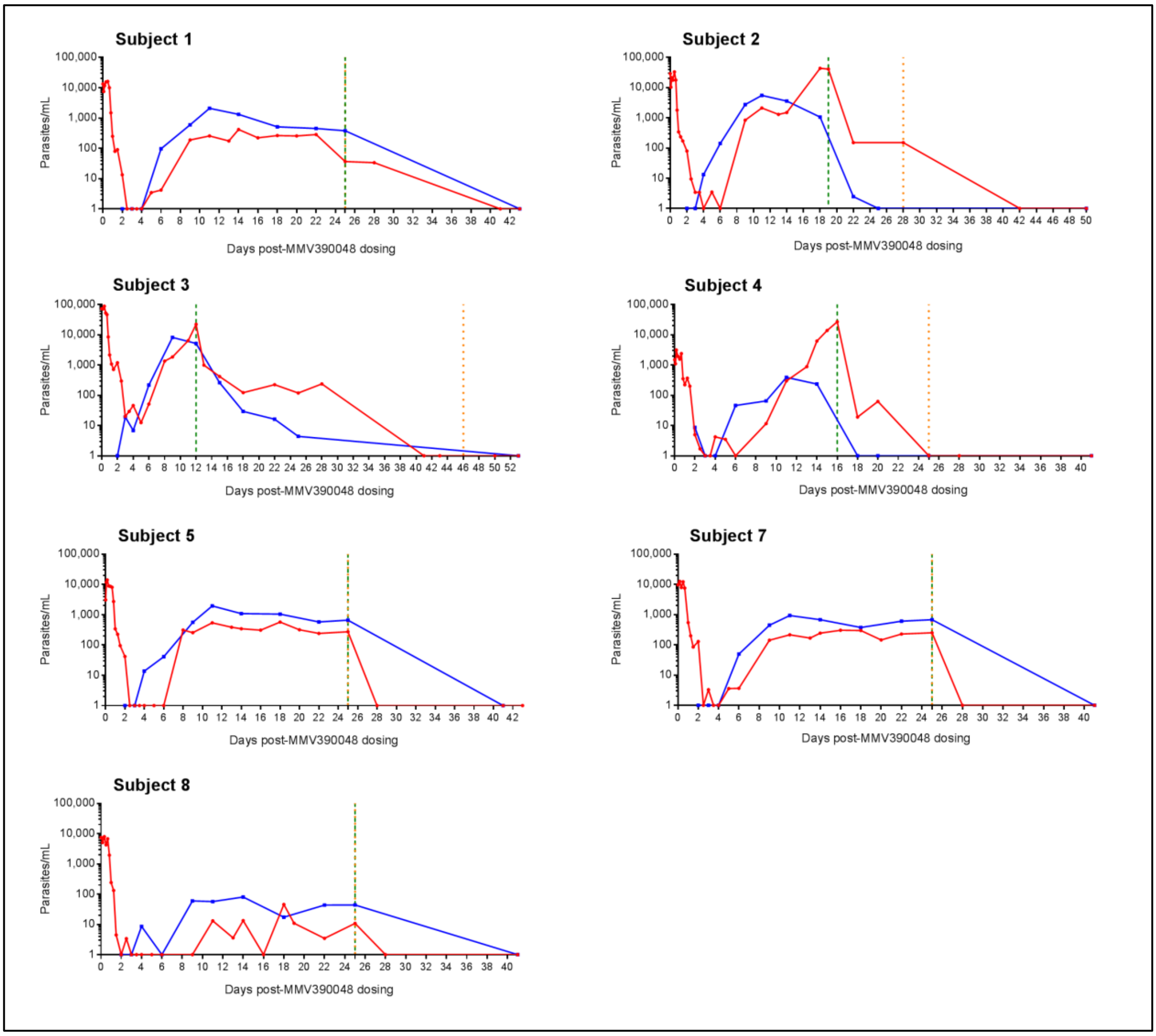

Figure S5. Individual subject parasitemia and gametocytemia profiles following dosing with 80 mg MMV390048. Total parasitemia measured by quantitative PCR targeting the gene encoding 18S rRNA is represented in red. Gametocytemia measured by quantitative reverse transcriptase PCR targeting *pfs25* mRNA is represented in blue. Green dashed lines represent time of treatment with artemether/lumefantrine. Orange dashed lines represent time of treatment with primaquine. Subjects 6 was not dosed with MMV390048 and was treated instead with artemether/lumefantrine.

Individual subject parasite clearance rates following MMV390048 treatment

Table S7. Individual $log_{10}PRR_{48}$ and parasite clearance half-life

Subject	$Log_{10}PRR_{48}$ (95%CI)	Parasite Clearance Half-Life (h) (95%CI)	P-value
**Cohort B1 (40 mg MMV390048)**			
Subject 1	2·1 (1·7–2·5)	6·9 (5·9–8·3)	$1{\cdot}94\times10^{-8}$
Subject 2	2·3 (1·9–2·8)	6·1 (5·3–7·4)	$8{\cdot}54\times10^{-8}$
Subject 3	3·3 (2·8–3·7)	4·4 (3·9–5·1)	$5{\cdot}79\times10^{-9}$
Subject 4	1·2 (0·8–1·7)	11·6 (8·4–18·6)	0·0001
Subject 5	2·5 (1·6–3·4)	5·8 (4·3–9·1)	0·0006
Subject 6	2·2 (1·9–2·5)	6·5 (5·7–7·6)	$1{\cdot}87\times10^{-9}$
Subject 7	2·3 (1·8–2·9)	6·2 (5·1–8·1)	$2{\cdot}25\times10^{-6}$
**Cohort B2 (80 mg MMV390048)**			
Subject 1	3·5 (2·9–4·2)	4·1 (3·5–4·9)	$4{\cdot}25\times10^{-7}$
Subject 2	2·4 (2·1–2·8)	6·0 (5·2–7·0)	$5{\cdot}60\times10^{-9}$
Subject 3	2·0 (1·6–2·3)	7·4 (6·3–8·9)	$4{\cdot}97\times10^{-8}$
Subject 4	2·4 (1·9–2·8)	6·1 (5·1–7·5)	$4{\cdot}19\times10^{-7}$
Subject 5	3·4 (2·9–3·9)	4·3 (3·7–5·0)	$4{\cdot}36\times10^{-7}$
Subject 7	3·1 (2·4–3·7)	4·7 (3·9–6·0)	$8{\cdot}11\times10^{-6}$
Subject 8	4·1 (3·1–5·1)	3·5 (2·8–4·6)	$1{\cdot}71\times10^{-5}$

$Log_{10}PRR_{48}$: logarithm to the base 10 of the parasite reduction ratio standardized over a 48 hour period after treatment; CI: confidence interval.

Safety results

Table S8. Summary of adverse events by dose cohort

	Single ascending dose study				Volunteer infection study	
**System Organ Class**<br>Preferred Term	**Placebo (n=6)**	**MMV390048 40 mg (n=6)**	**MMV390048 80 mg (n=6)**	**MMV390048 120 mg (n=6)**	**MMV390048 40 mg (n=7)**	**MMV390048 80 mg (n=8)**
	**n (%) M**					
Subjects with at least one adverse event	4 (66·7%) 13	0 (0·0%) 0	4 (66·7%) 9	5 (83·3%) 12	7 (100%) 99	8 (100%) 103
**Blood and lymphatic system disorders**	**0 (0·0%) 0**	**0 (0·0%) 0**	**0 (0·0%) 0**	**0 (0·0%) 0**	**4 (57·1%) 5**	**1 (12·5%) 1**
Lymphopenia	0 (0·0%) 0	0 (0·0%) 0	0 (0·0%) 0	0 (0·0%) 0	3 (42·9%) 3	1 (12·5%) 1
Neutropenia	0 (0·0%) 0	0 (0·0%) 0	0 (0·0%) 0	0 (0·0%) 0	2 (28·6%) 2	0 (0·0%) 0
**Gastrointestinal disorders**	**2 (33·3%) 4**	**0 (0·0%) 0**	**0 (0·0%) 0**	**2 (33·3%) 2**	**3 (42·9%) 7**	**5 (62·5%) 10**
Abdominal discomfort	0 (0·0%) 0	0 (0·0%) 0	0 (0·0%) 0	0 (0·0%) 0	3 (42·9%) 3	3 (37·5%) 4
Diarrhoea	1 (16·7%) 1	0 (0·0%) 0	0 (0·0%) 0	1 (16·7%) 1	1 (14·3%) 1	1 (12·5%) 1
Dyspepsia	0 (0·0%) 0	0 (0·0%) 0	0 (0·0%) 0	0 (0·0%) 0	0 (0·0%) 0	1 (12·5%) 1
Nausea	1 (16·7%) 2	0 (0·0%) 0	0 (0·0%) 0	1 (16·7%) 1	2 (28·6%) 2	2 (25·0%) 3
Vomiting	1 (16·7%) 1	0 (0·0%) 0	0 (0·0%) 0	0 (0·0%) 0	1 (14·3%) 1	1 (12·5%) 1
**General disorders and administration site conditions**	**2 (33·3%) 2**	**0 (0·0%) 0**	**1 (16·7%) 1**	**3 (50·0%) 3**	**6 (85·7%) 22**	**5 (62·5%) 24**
Chest discomfort	0 (0·0%) 0	0 (0·0%) 0	0 (0·0%) 0	1 (16·7%) 1	0 (0·0%) 0	0 (0·0%) 0
Chills	0 (0·0%) 0	0 (0·0%) 0	0 (0·0%) 0	0 (0·0%) 0	2 (28·6%) 3	2 (25·0%) 2
Fatigue	0 (0·0%) 0	0 (0·0%) 0	0 (0·0%) 0	0 (0·0%) 0	1 (14·3%) 1	4 (50·0%) 9
Malaise	0 (0·0%) 0	0 (0·0%) 0	0 (0·0%) 0	1 (16·7%) 1	5 (71·4%) 11	4 (50·0%) 7
Medical device site reaction	2 (33·3%) 2	0 (0·0%) 0	1 (16·7%) 1	1 (16·7%) 1	0 (0·0%) 0	0 (0·0%) 0
Pain	0 (0·0%) 0	0 (0·0%) 0	0 (0·0%) 0	0 (0·0%) 0	0 (0·0%) 0	1 (12·5%) 1
Pyrexia	0 (0·0%) 0	0 (0·0%) 0	0 (0·0%) 0	0 (0·0%) 0	4 (57·1%) 7	2 (25·0%) 5
**Infections and infestations**	**1 (16·7%) 1**	**0 (0·0%) 0**	**2 (33·3%) 3**	**1 (16·7%) 2**	**2 (28·6%) 2**	**0 (0·0%) 0**

	Single ascending dose study				Volunteer infection study	
**System Organ Class**<br>Preferred Term	**Placebo (n=6)**	**MMV390048 40 mg (n=6)**	**MMV390048 80 mg (n=6)**	**MMV390048 120 mg (n=6)**	**MMV390048 40 mg (n=7)**	**MMV390048 80 mg (n=8)**
Folliculitis	0 (0·0%) 0	0 (0·0%) 0	1 (16·7%) 1	1 (16·7%) 1	0 (0·0%) 0	0 (0·0%) 0
Herpes zoster	0 (0·0%) 0	0 (0·0%) 0	1 (16·7%) 1	0 (0·0%) 0	0 (0·0%) 0	0 (0·0%) 0
Upper respiratory tract infection	1 (16·7%) 1	0 (0·0%) 0	1 (16·7%) 1	1 (16·7%) 1	2 (28·6%) 2	0 (0·0%) 0
**Injury, poisoning and procedural complications**	**0 (0·0%) 0**	**0 (0·0%) 0**	**2 (33·3%) 2**	**0 (0·0%) 0**	**2 (28·6%) 2**	**2 (25·0%) 2**
Arthropod bite	0 (0·0%) 0	0 (0·0%) 0	0 (0·0%) 0	0 (0·0%) 0	1 (14·3%) 1	0 (0·0%) 0
Contusion	0 (0·0%) 0	0 (0·0%) 0	2 (33·3%) 2	0 (0·0%) 0	1 (14·3%) 1	1 (12·5%) 1
Laceration	0 (0·0%) 0	0 (0·0%) 0	0 (0·0%) 0	0 (0·0%) 0	0 (0·0%) 0	1 (12·5%) 1
**Investigations**	**0 (0·0%) 0**	**0 (0·0%) 0**	**0 (0·0%) 0**	**0 (0·0%) 0**	**0 (0·0%) 0**	**5 (62·5%) 17**
Alanine aminotransferase increased	0 (0·0%) 0	0 (0·0%) 0	0 (0·0%) 0	0 (0·0%) 0	0 (0·0%) 0	2 (25·0%) 3
Aspartate aminotransferase increased	0 (0·0%) 0	0 (0·0%) 0	0 (0·0%) 0	0 (0·0%) 0	0 (0·0%) 0	1 (12·5%) 1
Electrocardiogram pr prolongation	0 (0·0%) 0	0 (0·0%) 0	0 (0·0%) 0	0 (0·0%) 0	0 (0·0%) 0	1 (12·5%) 1
Lymphocyte count decreased	0 (0·0%) 0	0 (0·0%) 0	0 (0·0%) 0	0 (0·0%) 0	0 (0·0%) 0	4 (50·0%) 7
Neutrophil count decreased	0 (0·0%) 0	0 (0·0%) 0	0 (0·0%) 0	0 (0·0%) 0	0 (0·0%) 0	2 (25·0%) 3
White blood cell count decreased	0 (0·0%) 0	0 (0·0%) 0	0 (0·0%) 0	0 (0·0%) 0	0 (0·0%) 0	2 (25·0%) 2
**Metabolism and nutrition disorders**	**0 (0·0%) 0**	**0 (0·0%) 0**	**0 (0·0%) 0**	**0 (0·0%) 0**	**1 (14·3%) 1**	**3 (37·5%) 5**
Decreased appetite	0 (0·0%) 0	0 (0·0%) 0	0 (0·0%) 0	0 (0·0%) 0	1 (14·3%) 1	3 (37·5%) 5
**Musculoskeletal and connective tissue disorders**	**0 (0·0%) 0**	**0 (0·0%) 0**	**0 (0·0%) 0**	**0 (0·0%) 0**	**6 (85·7%) 21**	**7 (87·5%) 12**
Arthralgia	0 (0·0%) 0	0 (0·0%) 0	0 (0·0%) 0	0 (0·0%) 0	4 (57·1%) 6	1 (12·5%) 1
Back pain	0 (0·0%) 0	0 (0·0%) 0	0 (0·0%) 0	0 (0·0%) 0	1 (14·3%) 1	0 (0·0%) 0
Myalgia	0 (0·0%) 0	0 (0·0%) 0	0 (0·0%) 0	0 (0·0%) 0	5 (71·4%) 14	7 (87·5%) 11
**Nervous system disorders**	**1 (16·7%) 3**	**0 (0·0%) 0**	**1 (16·7%) 1**	**4 (66·7%) 5**	**7 (100%) 32**	**8 (100%) 29**
Dizziness	1 (16·7%) 1	0 (0·0%) 0	0 (0·0%) 0	0 (0·0%) 0	0 (0·0%) 0	0 (0·0%) 0
Headache	1 (16·7%) 2	0 (0·0%) 0	1 (16·7%) 1	4 (66·7%) 5	7 (100%) 30	8 (100%) 27
Lethargy	0 (0·0%) 0	0 (0·0%) 0	0 (0·0%) 0	0 (0·0%) 0	2 (28·6%) 2	1 (12·5%) 2
**Pregnancy, puerperium and perinatal conditions**	**1 (16·7%) 1**	**0 (0·0%) 0**	**0 (0·0%) 0**	**0 (0·0%) 0**	**0 (0·0%) 0**	**0 (0·0%) 0**
Abortion spontaneous	1 (16·7%) 1	0 (0·0%) 0	0 (0·0%) 0	0 (0·0%) 0	0 (0·0%) 0	0 (0·0%) 0
**Respiratory, thoracic and mediastinal disorders**	**0 (0·0%) 0**	**0 (0·0%) 0**	**0 (0·0%) 0**	**0 (0·0%) 0**	**3 (42·9%) 4**	**1 (12·5%) 1**

	Single ascending dose study				Volunteer infection study	
**System Organ Class** Preferred Term	**Placebo (n=6)**	**MMV390048 40 mg (n=6)**	**MMV390048 80 mg (n=6)**	**MMV390048 120 mg (n=6)**	**MMV390048 40 mg (n=7)**	**MMV390048 80 mg (n=8)**
Oropharyngeal pain	0 (0·0%) 0	0 (0·0%) 0	0 (0·0%) 0	0 (0·0%) 0	3 (42·9%) 4	1 (12·5%) 1
**Renal and urinary disorders**	**1 (16·7%) 1**	**0 (0·0%) 0**	**1 (16·7%) 1**	**0 (0·0%) 0**	**0 (0·0%) 0**	**0 (0·0%) 0**
Dysuria	0 (0·0%) 0	0 (0·0%) 0	1 (16·7%) 1	0 (0·0%) 0	0 (0·0%) 0	0 (0·0%) 0
Sterile pyuria	1 (16·7%) 1	0 (0·0%) 0	0 (0·0%) 0	0 (0·0%) 0	0 (0·0%) 0	0 (0·0%) 0
**Skin and subcutaneous tissue disorders**	**1 (16·7%) 1**	**0 (0·0%) 0**	**0 (0·0%) 0**	**0 (0·0%) 0**	**3 (42·9%) 3**	**2 (25·0%) 2**
Hyperhidrosis	0 (0·0%) 0	0 (0·0%) 0	0 (0·0%) 0	0 (0·0%) 0	2 (28·6%) 2	2 (25·0%) 2
Photosensitivity reaction	0 (0·0%) 0	0 (0·0%) 0	0 (0·0%) 0	0 (0·0%) 0	1 (14·3%) 1	0 (0·0%) 0
Rash	1 (16·7%) 1	0 (0·0%) 0	0 (0·0%) 0	0 (0·0%) 0	0 (0·0%) 0	0 (0·0%) 0
**Surgical and medical procedures**	**0 (0·0%) 0**	**0 (0·0%) 0**	**1 (16·7%) 1**	**0 (0·0%) 0**	**0 (0·0%) 0**	**0 (0·0%) 0**
Suture insertion	0 (0·0%) 0	0 (0·0%) 0	1 (16·7%) 1	0 (0·0%) 0	0 (0·0%) 0	0 (0·0%) 0

*If a subject had multiple occurrences of an adverse event, the subject is presented only once in the subject count (n) column for a given system organ class and preferred term. Occurrences are counted each time in the occurrence column (M).

Table S9. Alanine transaminase and aspartate transaminase values recorded during the study for the subject presenting with severe elevations

Day relative to dosing with 80 mg MMV390048	ALT value (U/L)	Normal/High/Low/ Clinically Significant Flag	AST value (U/L)	Normal/High/Low/ Clinically Significant Flag
-10	45	High, NCS	25	Normal
0	73	High, NCS	42	High, NCS
1	78	High, NCS	43	High, NCS
2	94	High, NCS	60	High, NCS
3	90	High, NCS	59	High, NCS
4	303	High, CS (7.6 ×ULN)	224	High, CS (5.6 ×ULN)
5	519	High, CS (13.0 ×ULN)	319	High, CS (8.0 ×ULN)
6	518	High, CS (13.0 ×ULN)	237	High, CS (5.9 ×ULN)
9	379	High, CS (9.5 ×ULN)	107	High, NCS
13	204	High, CS (5.1 ×ULN)	51	High, NCS
18	168	High, CS (4.2 ×ULN)	110	High, NCS
19	150	High, CS (3.8 ×ULN)	113	High, NCS
22	100	High, NCS	44	High, NCS
28	105	High, NCS	32	Normal
42	61	High, NCS	47	High, NCS
50	27	Normal	24	Normal

ALT: alanine aminotransferase; AST: aspartate aminotransferase; NCS: non-clinically significant; CS: clinically significant; ×ULN: times upper limit of normal.
Laboratory normal reference ranges: ALT 5-40 U/L, AST 10-40 U/L.

MMV390048 resistance assessment methods and results

Methods

Blood samples were collected from subjects at the time when parasites recrudesced for DNA sequencing to determine whether the increase in parasitemia was due to the emergence of a population with a resistance-conferring mutation in the *P. falciparum* PI4K gene (PlasmoDB accession PF3D7_0509800). Samples were sequenced by PCR amplification of the PI4K gene using KAPA Hifi Readymix PCR Kit. The gene was split into 4 overlapping sections, and each section was amplified through 2 rounds of nested PCR using internal and external primers. Sanger sequencing was performed by Genewiz LLC and the sequences were analysed and aligned using DNAStar's SeqMan Pro program.

Table S10. *S*equencing primers and protocols for each nested PCR reaction

Region*	External and internal primers name (P number) and sequence	PCR Protocol		
**Region 1**				
**External 1392bp**	P7142 GTTGACATTGAAAATAGTCAACATCC	92°C	0:45	
		92°C	0:24	×30
	P7143 TCAATTTCTTGTATCATTCGTTGAGC	54°C	0:24	
		62°C	1:30	
		62°C	4:00	
**Internal 1354bp**	P7144 GGAAGGAGTAAAAGATGAGGGGG	92°C	0:45	
		92°C	0:24	×35
	P7145 GCAAAATGCATTGACTTACTAGC	57°C	0:24	
		62°C	1:30	
		62°C	4:00	
**Region 2**				
**External 1374bp**	P7146 TATGATAGAAAAGAAGTTGGTGTAC	92°C	0:45	
		92°C	0:24	×30
	P7147 GTGACTAATATCTTCATCCAAATCAGC	54°C	0:24	
		62°C	1:30	
		62°C	4:00	
**Internal 1136 bp**	P7148 CTTTAGTAAGATATGAATCTTCATCTC	92°C	0:45	
		92°C	0:24	×35
	P7149 ATTTGTATCATGATTAAATGTTGAGG	57°C	0:24	
		62°C	1:30	
		62°C	4:00	
**Region 3**				
**External 1461bp**	P7150 TGAATATGAGAAGATGTATAGTGGC	92°C	0:45	
		92°C	0:24	×30
	P7151 CAGGTTTAAAATATTCACTCAGATC	54°C	0:24	
		62°C	1:30	
		62°C	4:00	
**Internal 1356bp**	P7152 TACCATTAGAAAGTATGTCTTCCTCA	92°C	0:45	
		92°C	0:24	×35
	P7153 GAAGTTACTAATGGTATTATCTTGG	57°C	0:24	
		62°C	1:30	
		62°C	4:00	
**Region 4**				
**External 1425bp**	P7154 TTATACAACAACAAATCACAGAGGG	92°C	0:45	
		92°C	0:24	×30
	P7155 TTTGAAAGTAGTCATACTGGACAC	50°C	0:24	
		62°C	1:30	
		62°C	4:00	
**Internal 1364bp**	P7156 TTTGAAAGTAGTCATACTGGACAC	92°C	0:45	
		92°C	0:24	×35
	P7157 TCGAAAATTATTAATGGACGCTTC	57°C	0:24	
		62°C	1:30	
		62°C	4:00	

*The expected length of the amplified sequence in base pairs (bp) is provided for each internal and external region.

Results

No mutations in the *Plasmodium* PI4K gene were detected in any of the samples analysed (Table S11). Previous *in vitro* studies have shown the development of resistance to *Plasmodium* PI4K inhibitors through copy number variation and selection of single nucleotide polymorphisms (SNPs) [2, 3]. The SNPs that arose in response to selection under drug pressure were S743T, A1319V, S1320L, Y1356F, and H1484Y [2, 3]. Many of these resistance-conferring mutations mapped to the catalytic domain of *P. falciparum* PI4K, but neither these nor any novel mutations were found in the samples analyzed. Despite repeated efforts to optimize the PCR, the reactions to amplify the first region did not yield any PCR product. Therefore, samples were sequenced starting at codon 427; however, no previous studies reported any mutations in this early part of the gene.

Table S11. *Plasmodium* PI4K gene sequencing

Subject	Study day*	Time post-MMV390048 dosing	Sample volume (µl)	Estimated number of parasites in sample	Mutations in *Plasmodium* PI4K
**Cohort B1 (40 mg MMV390048)**					
Subject 1	Day 23	15 days	15	1373	None
Subject 2	Day 21	13 days	30	1077	None
Subject 4	Day 15	7 days	10	3165	None
Subject 5	Day 10	2 days	15	1071	None
Subject 6	Day 18	10 days	50	951	None
Subject 7	Day 19	11 days	10	2588	None
**Cohort B2 (80 mg MMV390048)**					
Subject 2	Day 26	18 days	10	2187	None
Subject 3	Day 19	11 days	15	479	None
Subject 4	Day 24	16 days	10	1182	None

*Day 0 = inoculation day.

Malaria transmission results

Table S12. Membrane feeding assays

**Subject**	**Feeding day**	**Feeding rate (no. fed mosquitoes/ total no. mosquitoes [%])**	**Mortality rate (no. dead mosquitoes/ total no. mosquitoes [%])**	**Infection rate (no. mosquitoes with oocysts/no. mosquitoes examined [%])**	**Gametocytemia on feeding day (gametocytes/ml of blood)**
**Cohort B1 (40 mg MMV390048)**					
Subject 1	Day 15	79/81 (97·5%)	10/81 (12·3%)	1/50 (2·0%)	4541
Subject 3	Day 15	76/76 (100·0%)	8/76 (10·5%)	0/50 (0·0%)	4195
**Cohort B2 (80 mg MMV390048)**					
Subject 1	Day 14	98/98 (100·0%)	15/98 (15·0%)	0/83 (0·0%)	1326
Subject 2	Day 14	108/110 (98·2%)	34/110 (34·6%)	3/76 (3·9%)	3600
Subject 3	Day 12	105/113 (92·9%)	21/113 (18·6%)	10/92 (10·9%)	5201
Subject 5	Day 14	105/106 (99·1%)	38/106 (38·4%)	1/68 (1·5%)	1099

References

1. Llanos-Cuentas A, Casapia M, Chuquiyauri R, Hinojosa JC, Kerr N, Rosario M, et al. Antimalarial activity of single-dose DSM265, a novel *Plasmodium* dihydroorotate dehydrogenase inhibitor, in patients with uncomplicated *Plasmodium falciparum* or *Plasmodium vivax* malaria infection: a proof-of-concept, open-label, phase 2a study. *Lancet Infect Dis*. 2018;**18**: 874-883.
2. McNamara CW, Lee MC, Lim CS, Lim SH, Roland J, Simon O, et al. Targeting *Plasmodium* PI(4)K to eliminate malaria. *Nature*. 2013;**504**: 248-253.
3. Paquet T, Le Manach C, Cabrera DG, Younis Y, Henrich PP, Abraham TS, et al. Antimalarial efficacy of MMV390048, an inhibitor of *Plasmodium* phosphatidylinositol 4-kinase. *Sci Transl Med*. 2017;**9**: eaad9735.
